# A Piecewise Linearization Based Method for Crossed Frequency Admittance Matrix Model Calculation of Harmonic Sources

**DOI:** 10.3390/s25020582

**Published:** 2025-01-20

**Authors:** Youhang Yang, Shaorong Wang, Mingming Shi, Xian Zheng

**Affiliations:** 1 School of Electrical and Electronic Engineering, Huazhong University of Science and Technology, Wuhan 430074, China; d202180642@hust.edu.cn; 2 Electric Power Research Institute of State Grid Jiangsu Electric Power Company, Nanjing 211103, China; simon8612@126.com (M.S.); scuzxx@163.com (X.Z.)

**Keywords:** power quality, harmonic source model, phase-controlled power electronic equipment, piecewise linearization, crossed frequency admittance matrix, analytical model

## Abstract

The integration of large-scale power electronic equipment has intensified harmonic issues in power systems. Accurate harmonic models are fundamental for evaluating and mitigating harmonic problems, but existing models still exhibit deficiencies in harmonic mechanism, model complexity and accuracy. This work proposes a calculation method of crossed frequency admittance matrix (CFAM) analytical model based on piecewise linearization, aiming to achieve accurate modeling of phase-controlled power electronic harmonic sources. Firstly, the traditional CFAM model construction methods are introduced, and the shortcomings in harmonic modeling are discussed. Subsequently, the parameter-solving process of the CFAM analytical model based on piecewise linearization is proposed. This method improves the accuracy of harmonic modeling and simplifies the construction process of the analytical model. Furthermore, taking single-phase and three-phase bridge rectifiers as examples, CFAM analytical models under intermittent and continuous load current conditions are established, respectively, and the unified harmonic models for both conditions are summarized. Finally, case studies of rectifier harmonic sources under varying circuit control parameters and supply voltage distortions are conducted through Matlab/Simulink and experiments. The results demonstrate that the proposed method provides higher accuracy and more stable performance for harmonic current estimation compared with the traditional CFAM model and constant current source model.

## 1. Introduction

With the transformation of energy structure and the promotion of new power system construction, the application of power electronic equipment in generation, transmission, distribution, and consumption has become increasingly widespread [1,2,3]. As typical nonlinear loads in power systems, the operating characteristics of power electronic equipment lead to distortion of voltage and current waveforms, making harmonic issues more complex [4,5]. Harmonics not only degrade power quality but also increase losses in transmission lines and electrical equipment [6,7]. In severe cases, harmonics even cause equipment failures, threatening the safe and stable operation of power systems [8,9].

Switching devices commonly used in power electronic equipment include power diodes, thyristors, insulated-gate bipolar transistors (IGBTs), etc. According to different types of switching devices, power electronic equipment can be divided into three categories: uncontrolled, semi-controlled and fully controlled [10,11]. Among them, semi-controlled equipment adopts phase-control methods, while uncontrolled equipment can be considered a special case of phase-controlled equipment with a trigger angle of zero. Fully controlled equipment usually utilizes pulse width modulation (PWM) technology, which mainly generates switching frequency doubling and sideband harmonics during operation, with relatively low harmonic content [12,13]. In contrast, uncontrolled and semi-controlled equipment produce higher harmonic content and are the primary power electronic harmonic sources, which are widely used in distributed loads such as household appliances and electric vehicle charging piles [14,15], as well as high-power loads like electrolytic aluminum rectifier power supply systems [16], and also used in HVDC transmission fields such as LCC-HVDC and UHVDC [17,18].

Establishing accurate harmonic models and exploring the mechanism of harmonics are crucial for assessing and mitigating harmonic issues, which also serve as the foundation for harmonic power flow calculation [19,20], harmonic contribution estimation and other related harmonic analysis work [21,22]. Currently, scholars have conducted extensive research on harmonic source modeling, which can be broadly categorized into two types: mechanism modeling and data-driven modeling.

The mechanism modeling method establishes analytical expressions among currents and voltages of different orders at the point of common coupling (PCC) based on the operation process of harmonic sources. The constant current source (CCS) model has been proposed in [23], which utilizes the typical harmonic spectrum to replace each order current as a separate current source. The CCS model is structurally simple and convenient for application, but it fails to account for the influence of voltage variations on harmonic currents, thus limiting its accuracy. Reference [24] extends the CCS model by adding an admittance branch, proposing the Norton model for harmonic sources. The Norton model can reflect the coupling effect of the same-order PCC voltage on harmonic currents, but the influence of non-identical order harmonic voltages is not considered. Based on the small signal theory, the harmonic source is linearized around the operating point and the crossed frequency admittance matrix (CFAM) harmonic model is proposed in [25]. It also presents a method for calculating the CFAM by sequentially applying each order voltage to the harmonic source. The CFAM model considers the coupling relationship among voltages and currents of different orders but may introduce significant errors when harmonic sources deviate far from the operating point. Reference [26] uses switching functions to simulate the on-off state of switching devices. Through complex mathematical derivation and induction, CFAM analytical models of the single-phase bridge rectifier under continuous load current conditions are established. Every matrix parameter of CFAM in this model is the sum of an infinite series, which inevitably has truncation errors in practical application.

The data-driven modeling method utilizes measured or simulated data to develop external characteristic models of harmonic sources with the help of intelligent algorithms such as probability statistics and machine learning. In [27,28], radial basis function neural networks have been employed to establish the mapping relationship among currents and voltages of different orders, as well as load characteristic parameters for the three-phase PWM voltage source inverters. A combination of recursive partial least squares and adaptive updating strategies is proposed in [29] for dynamic assessment of harmonics generated by household appliances. Reference [30] proposes a harmonic modeling method based on long short-term memory (LSTM) neural networks to capture the coupling relationship between harmonic voltages and currents. From the perspective of probability statistics, a general harmonic probability model was constructed in [31] by fitting the actual probability distribution of harmonic currents using a variety of typical probability density functions. Although the above data-driven models have made some progress in harmonic source modeling, there are still some shortcomings: firstly, the interpretability of data-driven models is poor, and the parameters lack physical significance, which hinders in-depth analysis of the harmonic generation mechanisms; secondly, the selection of datasets is difficult to cover various operating conditions of harmonic sources, which limits the application in harmonic power flow calculation.

In this context, this paper focuses on phase-controlled power electronic harmonic sources and proposes a calculation method for the CFAM analytical model based on piecewise linearization. Before and after the conduction of switching devices, phase-controlled equipment can be equivalent to linear circuits in different operating stages. The piecewise linearization method is employed to individually investigate the harmonic currents induced by any harmonic voltage at PCC, thereby deriving the CFAM analytical model for harmonic sources. Taking single-phase and three-phase bridge rectifiers as examples, CFAM analytical models are developed separately for intermittent and continuous load current conditions, and the unified harmonic models for both conditions are further established through generalization. Simulations with variable parameters are conducted using Matlab/Simulink software, which verifies the effectiveness and accuracy advantages of the proposed harmonic modeling method. The main contributions of this paper are as follows:

(1) This paper proposes a calculation method of the CFAM analytical model for phase-controlled power electronic harmonic sources. The method improves the precision of harmonic source modeling and simplifies the construction process of the analytical model through piecewise linearization. The constructed CFAM analytical model can accurately reflect the coupling relationship between voltages and currents of different orders.
(2) The CFAM analytical models of the single-phase and three-phase bridge rectifiers under intermittent and continuous load current conditions are established using the proposed method, respectively. The Fourier coefficients of the CFAM analytical models exhibit regular cosine and sine symmetry under both conditions, which streamlines the derivation process of the harmonic model and facilitates programming calculations.
(3) The unified harmonic models for the single-phase and three-phase bridge rectifiers under both operating conditions are, respectively, developed through induction. Extensive case studies under different circuit control parameters and supply voltage distortions are conducted, verifying the effectiveness of the proposed method and the accuracy advantages of the CFAM analytical model.

The remainder of this paper is organized as follows. Section 2 introduces the traditional CFAM model construction methods and analyzes its shortcomings in harmonic modeling. On this basis, Section 3 presents the proposed parameter-solving approach for the CFAM analytical model. Taking single-phase and three-phase bridge rectifiers as examples, the unified CFAM analytical models under intermittent and continuous load current conditions are established, respectively. Case studies are carried out in Section 4 to verify the effectiveness of the proposed method and demonstrate precision advantage compared to the traditional CFAM model and CCS model. Section 5 concludes this paper.

## 2. Traditional Crossed Frequency Admittance Matrix Model

### 2.1. CFAM Model Based on Small Signal Theory

There exists a functional relationship between the harmonic current generated by the nonlinear load and the PCC voltages of different orders [23]. According to the small signal theory, the function can be linearized around a certain steady-state operating point. The concept of CFAM is proposed in [25] to characterize the linear coupling relationship among the harmonic current and different order voltages, as shown in  (1).

(1) $${\dot{I}}_{s}=Y{\dot{U}}_{s}$$

where ${\dot{I}}_{s},{\dot{U}}_{s}\in R^{N_{h}\times 1}$, $Y\in R^{N_{h}\times N_{h}}$, $N_{h}$ is the total number of harmonics considered; ${\dot{I}}_{s}$ and ${\dot{U}}_{s}$ denote the phasor matrices of different order currents and voltages at PCC, respectively; Y represents the CFAM of the harmonic source, and its matrix element $Y_{hk}$ denotes the first-order partial derivative of the *h*-th current with respect to the *k*-th voltage. As noted in [25], since $Y_{hk}$ corresponds to the ratio of current and voltage at different orders, it is called “crossed frequency admittance” for convenience. Additionally, a method for calculating the harmonic source CFAM based on small signal operations is provided in [25], which mainly consists of the following two steps:

(1) The fundamental voltage is applied to establish the steady-state operating point of the harmonic source. Voltage and current transformers are utilized for measurement at PCC, and the collected data are processed using the Fast Fourier Transform (FFT). Subsequently, the first column elements of CFAM can be calculated according to (2).
  (2) $$Y_{h1}={\dot{I}}_{\mathrm{ref}\;h}/{\dot{V}}_{\mathrm{ref}\;1},\;h=1,2,\cdots ,H\;$$
  where ${\dot{V}}_{\mathrm{ref}\;1}$ denotes the applied fundamental voltage, and ${\dot{I}}_{\mathrm{ref}\;h}$ represents the *h*-th current at PCC.
(2) Keeping the fundamental voltage constant, the *k*-th harmonic voltage is superimposed sequentially to linearize the harmonic source around the steady-state operating point. Relative to the case where only the fundamental voltage is applied, the fluctuations in different order currents at PCC are assumed to be caused solely by the *k*-th harmonic voltage. Consequently, the element $Y_{hk}$ of CFAM can be calculated in columns by (3).
  (3) $$Y_{hk}=\frac{{\dot{I}}_{sh}-{\dot{I}}_{\mathrm{ref}\;h}}{{\dot{V}}_{sk}-{\dot{V}}_{\mathrm{ref}\;k}},\;k=2,3,\cdots ,K\;$$
  where ${\dot{V}}_{\mathrm{ref}\;k}$ represents the *k*-th harmonic voltage at PCC when only the fundamental voltage is applied; ${\dot{I}}_{sh}$ and ${\dot{V}}_{sk}$, respectively, denote the *h*-th harmonic current and the *k*-th harmonic voltage after performing the small signal operation.

From the above calculation process, it can be observed that the CFAM model based on small signal theory relies on the steady-state operating point of the harmonic source. When the operating state of the harmonic source deviates from the steady-state point, the CFAM model may exhibit considerable errors. Furthermore, this CFAM calculation method is suitable for experiments with low-power harmonic sources and software simulations, but challenging to implement for harmonic sources in operation.

### 2.2. CFAM Model Utilizing Switching Function Method

Power electronic equipment precisely regulates and converts electrical energy by periodically turning on and off the circuit through switching devices. Based on modulation theory, switching functions can be used to simulate the on and off states of switching devices. Taking the rectifier as an example, the main process of establishing the harmonic source CFAM model based on the switching function method is introduced below.

The DC side voltage $v_{\mathrm{dc}}$ of the rectifier can be obtained by modulating the AC side voltage $v_{\mathrm{ac}}$ with the switching function $S_{v}$, as shown in  (4).

(4) $$v_{\mathrm{dc}}\left(t\right)=S_{v}\left(t\right)\cdot v_{\mathrm{ac}}\left(t\right)\;$$

**Figure 1:**
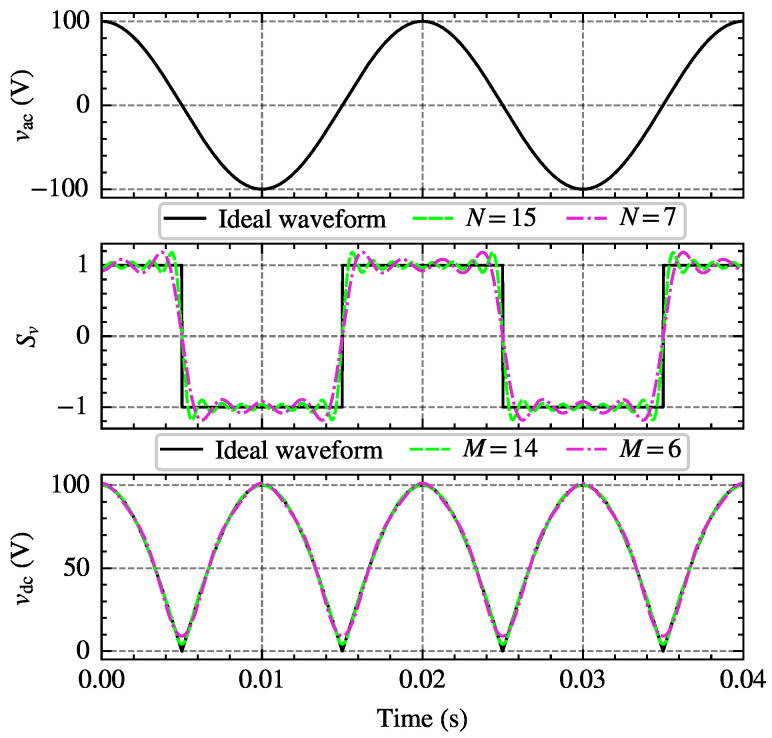
Modulation process of DC side voltage.

$S_{v}$ can be represented using a Fourier series, while $v_{\mathrm{ac}}$ is a set of sine and cosine functions containing the fundamental voltage. By appropriately transforming the product of the two terms through trigonometric transformations, the frequency-domain expression of $v_{\mathrm{dc}}$ can be obtained. Figure 1 illustrates the process of obtaining DC voltage by modulating AC voltage with a switching function. Here, $v_{\mathrm{ac}}\left(t\right)=\sqrt{2}V_{1}\cos\left(\omega t\right)$, and $S_{v}$ is given by (5).

(5) $$S_{v}\left(t\right)=\left\{\begin{array}{cc} -1, & \mathrm{if}\;T/4+kT<t<3T/4+kT\\ 1, & \mathrm{if}\;3T/4+kT<t<5T/4+kT \end{array}\right.=\frac{4}{\pi }\sum_{n=1,3,5}^{N}{\left(-1\right)}^{\frac{n-1}{2}}\frac{1}{n}\cos n\omega t$$

where *T* is the period of the fundamental voltage, and *k* is a non-negative integer. By substituting $v_{\mathrm{ac}}$ and $S_{v}$ into (4), the DC side voltage $v_{\mathrm{dc}}$ can then be obtained through trigonometric transformations, as shown in (6).

(6) $$v_{\mathrm{dc}}\left(t\right)=\frac{4\sqrt{2}V_{1}}{\pi }\sum_{m=2,4,6}^{N-1}\frac{{\left(-1\right)}^{\frac{m}{2}-1}\cos m\omega t}{\left(m-1)(m+1\right)}+\frac{2\sqrt{2}V_{1}}{\pi }\;$$

If the load impedance on the DC side is $Z_{\mathrm{dc}}$, calculations can be performed using the phasor method, and then the voltage and current are converted into trigonometric form. The current $i_{\mathrm{dc}}$ flowing through the load can be calculated by:

(7) $$i_{\mathrm{dc}}\left(t\right)=v_{\mathrm{dc}}\left(t\right)/Z_{\mathrm{dc}}\;$$

The AC side current of the rectifier can be obtained by modulating the DC side current $i_{\mathrm{dc}}$ with the switching function $S_{i}$, as shown in  (8).

(8) $$i_{\mathrm{ac}}\left(t\right)=S_{i}\left(t\right)\cdot i_{\mathrm{dc}}\left(t\right)\;$$

Based on the derivation of (4)–(8), the expressions for the voltage and current on the AC side of the rectifier are obtained. Furthermore, through extensive formula derivation and induction, reference [26] establishes the CFAM analytical model for the single-phase bridge rectifier under continuous current conditions using the switching function method, as illustrated in (9).

(9) $${\dot{I}}_{s}=Y^{+}{\dot{U}}_{s}+Y^{-}{\hat{U}}_{s}\;$$

where ${\hat{U}}_{s}$ is the matrix of different order voltage conjugate phasors at PCC; $Y^{+},Y^{-}\in R^{N_{h}\times N_{h}}$, whose matrix elements denote the coupling admittances between current phasors and voltage phasors, as well as between current phasors and conjugate voltage phasors.

Each matrix element of the CFAM in (9) is a superposition of infinite series. The more terms are accumulated, the more accurate the harmonic source modeling result, but this also significantly increases the computational burden. Consequently, when using the switching function method for harmonic source modeling in practical applications, truncation errors are inevitably introduced. Comparing (1) and (9), it can be observed that the number of CFAM elements in the latter is twice that of the former. When the power electronic equipment is operated under intermittent current conditions, one set of switching devices completely turns off before another set begins to conduct. In harmonic analysis that includes dynamic processes, the accuracy of the CFAM model established using the switching function method will be limited. Furthermore, under intermittent current conditions, the conduction angle of the switching devices is influenced by PCC voltages of different orders. This complicates the accurate formulation of the switching function, making it challenging to derive the CFAM model in the form of (9).

## 3. CFAM Analytical Model for Harmonic Sources Based on Piecewise Linearization

### 3.1. Parameter-Solving Process of CFAM Analytical Model

Due to the combined effects of background harmonic voltages in the power system and those generated by power electronic equipment, PCC voltages are no longer pure sinusoidal waves but contain a series of harmonics. Directly using the full voltage for harmonic source modeling would significantly increase the computational complexity.

**Figure 2:**
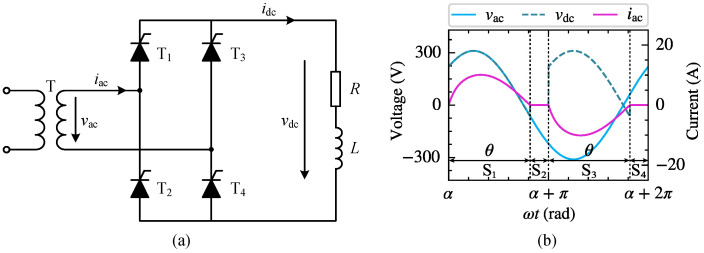
Single-phase bridge-controlled rectifier circuit topology (**a**) and corresponding schematic waveforms under intermittent load current conditions (**b**).

Under normal operating conditions, phase-controlled power electronic equipment periodically switches the current on and off. Before and after the switching devices conduct, the phase-controlled power electronic equipment can be equivalently considered as a linear circuit operating in different stages. Taking the single-phase bridge-controlled rectifier in Figure 2 as an example for illustration, α and θ represent the trigger and conduction angles of the thyristor, respectively. When $\omega t\in \left[\alpha ,\alpha +\theta \right]$, thyristors $T_{1}$ and $T_{4}$ conduct, and the DC side load is supplied by $v_{\mathrm{ac}}$, denoted as circuit state $S_{1}$; when $\omega t\in \left[\alpha +\theta ,\alpha +\pi \right]$, thyristors $T_{1}$ and $T_{4}$ are off, and the loop current is 0, denoted as circuit state $S_{2}$; when $\omega t\in \left[\alpha +\pi ,\alpha +\pi +\theta \right]$, thyristors $T_{2}$ and $T_{3}$ conduct, and the DC side load is supplied by $-v_{\mathrm{ac}}$, denoted as circuit state $S_{3}$; when $\omega t\in \left[\alpha +\pi +\theta ,\alpha +2\pi \right]$, thyristors $T_{2}$ and $T_{3}$ are off, denoted as circuit state $S_{4}$. From the perspective of the entire fundamental period, the single-phase bridge-controlled rectifier exhibits nonlinear characteristics. However, for the individual operating stage $S_{1}$ to $S_{4}$, the rectifier can be treated as a linear circuit. Consequently, when modeling the harmonic sources of phase-controlled power electronic equipment, it is feasible to separately investigate the currents induced by the *k*-th harmonic voltage $v_{k}$ at PCC for different operating stages. By further decomposition using the Fourier series, the different order currents induced by $v_{k}$ can be obtained.

**Figure 3:**
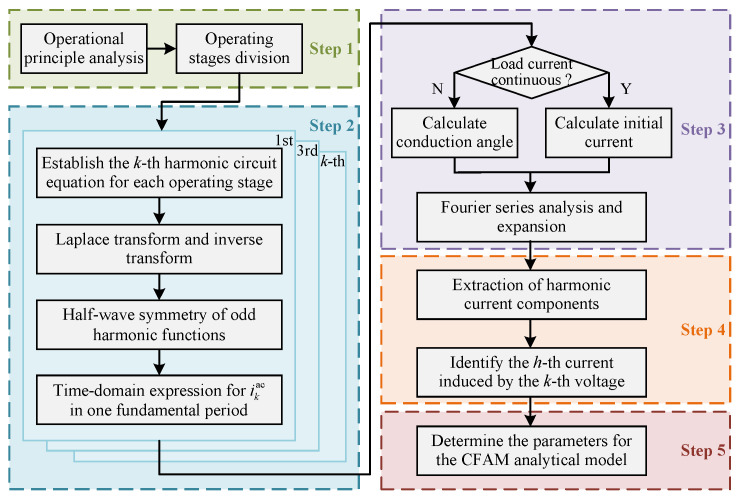
Establishment process of CFAM analytical model based on piecewise linearization.

Based on the piecewise linearization method, the parameter-solving process for the CFAM analytical model of the phase-controlled power electronic equipment proposed in this paper is illustrated in Figure 3. The main steps are summarized as follows:

(1) Division of operating stages. Analyze the operational principle of the phase-controlled power electronic equipment and determine the operating stages within one fundamental period based on the conduction sequence of the switching devices, denoted as $S_{1},S_{2},\ldots ,S_{2m}$. Subsequently, the piecewise linearization method is applied to establish circuit equations for each operating stage, corresponding to the *k*-th harmonic contained in PCC voltages.
(2) Derivation of time-domain current expressions. The voltages at PCC are odd harmonic functions with half-wave symmetry [32]. Consequently, it is sufficient to calculate the AC side current induced by the *k*-th voltage in half fundamental period and then obtain the time-domain expression for the current over a full fundamental period through translation and flipping, as expressed in (10).
  (10) $$i_{k}^{\mathrm{ac}}\left(t\right)=\left\{\begin{array}{cc} f_{1}\left(t\right), & \omega t\in S_{1}\\ \cdots & \cdots \\ f_{m}\left(t\right), & \omega t\in S_{m}\\ -f_{1}\left(t-\pi /\omega \right), & \omega t\in S_{m+1}\\ \cdots & \cdots \\ -f_{m}\left(t-\pi /\omega \right), & \omega t\in S_{2m} \end{array}\right.$$
  where $f_{m}\left(t\right)$ represents the AC side current expression of the phase-controlled power electronic equipment at the *m*-th operating stage.
(3) Fourier series analysis and expansion. Fourier series decomposition is performed on $i_{k}^{\mathrm{ac}}$, which can be expanded into the trigonometric function form shown in (11).
  (11) $$i_{k}^{\mathrm{ac}}\left(t\right)=\frac{V_{k\mathrm{dc}}}{\left|Z_{k}\right|}\left[\frac{C_{0}}{2}+\sum_{h=1}^{H}\left(a_{h}\cos h\omega t+b_{h}\sin h\omega t\right)\right]\;$$
  where $C_{0}$, $a_{h}$, $b_{h}$ are the coefficients of the Fourier series, respectively, and due to the half-wave symmetry of the odd harmonic function, the DC component $C_{0}$ equals 0; $V_{k\mathrm{dc}}$ represents the peak voltage of the DC side load, and $Z_{k}$ is the *k*-th harmonic impedance of the power electronic equipment after the switching devices are turned on.
(4) Extraction of harmonic current components. Based on the result of Fourier series decomposition, the *h*-th current $i_{hk}$ caused by the *k*-th voltage $v_{k}$ is further extracted, and the phasor form can be expressed as:
  (12) $${\dot{I}}_{hk}=\frac{V_{k\mathrm{dc}}}{\sqrt{2}\left|Z_{k}\right|}\left(a_{h}∠\frac{\pi }{2}+b_{h}∠0\right)\;$$
(5) Determination of CFAM analytical model parameters. This paper utilizes the CFAM model described in (1), with the matrix form presented in (13). The CFAM element $Y_{hk}$ represents the coupling relationship between the *h*-th current and the *k*-th voltage and the analytical expression for $Y_{hk}$ as proposed in this paper is presented in (14).
  (13) $$\left[\begin{array}{c} {\dot{I}}_{s1}\\ {\dot{I}}_{s2}\\ \vdots \\ {\dot{I}}_{sH} \end{array}\right]=\left[\begin{array}{cccc} Y_{11} & Y_{12} & \cdots & Y_{1H}\\ Y_{21} & Y_{22} & \cdots & Y_{2H}\\ \vdots & \vdots & \ddots & \vdots \\ Y_{H1} & Y_{H2} & \cdots & Y_{HH} \end{array}\right]\left[\begin{array}{c} {\dot{V}}_{s1}\\ {\dot{V}}_{s2}\\ \vdots \\ {\dot{V}}_{sH} \end{array}\right]$$
  (14) $$Y_{hk}=\frac{{\dot{I}}_{hk}}{{\dot{V}}_{k}}=\frac{V_{k\mathrm{dc}}}{V_{km}\left|Z_{k}\right|}\left[a_{h}∠\left(\frac{\pi }{2}-\varphi_{k}\right)+b_{h}∠\left(-\varphi_{k}\right)\right]$$
  where ${\dot{I}}_{s1},{\dot{I}}_{s2},\ldots ,{\dot{I}}_{sH}$ represent the different order currents on the AC side of the harmonic source; ${\dot{V}}_{s1},{\dot{V}}_{s2},\ldots ,{\dot{V}}_{sH}$ denote the different order voltages at PCC; ${\dot{V}}_{k}$ is the phasor form of $v_{k}$; $V_{km}$ and $\varphi_{k}$ are the amplitude and phase of $v_{k}$, respectively.

Employing the piecewise linearization method aids in studying the generation mechanism of harmonic currents, while simultaneously simplifying the complexity of harmonic source modeling. Furthermore, the derivation process demonstrates that the CFAM calculated using the proposed method effectively avoids truncation errors. To further illustrate the modeling process and effectiveness of the proposed method, the CFAM analytical models for the single-phase and three-phase bridge rectifiers under intermittent and continuous load current conditions are derived in Section 3.2 and Section 3.3, respectively.

### 3.2. Establishment of CFAM Analytical Model for the Single-Phase Bridge Rectifier

**Figure 4:**
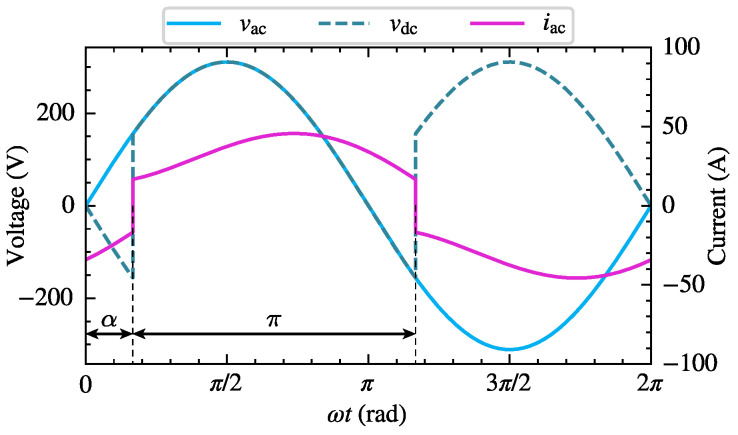
Schematic waveforms of AC side voltage $v_{\mathrm{ac}}$, DC side voltage $v_{\mathrm{dc}}$, and AC side current $i_{\mathrm{ac}}$ of the single-phase bridge-controlled rectifier under continuous load current conditions.

The single-phase bridge rectifier is a common nonlinear harmonic source in household appliances and small industrial equipment. Considering that uncontrolled power electronic equipment can be regarded as a special case of phase-controlled power electronic equipment with a zero trigger angle, this section takes the single-phase bridge-controlled rectifier circuit shown in Figure 2a as an example to establish its CFAM model based on the proposed method. The load impedance angle and thyristor trigger angle α have a significant impact on the operating characteristics of the circuit. When the load impedance angle is small and α is large, the DC side load current is intermittent. Conversely, if the load impedance angle is large and α is small, the load current is continuous. Figure 2b and Figure 4 demonstrate the schematic waveforms of the AC side voltage, current and DC side voltage of the rectifier circuit under intermittent and continuous load current conditions, respectively. It can be observed that the current waveforms differ significantly under the two conditions due to the different operating stages involved. Consequently, in order to accurately study the harmonic generation mechanism of the single-phase bridge-controlled rectifier, it is necessary to establish corresponding harmonic models for both conditions, respectively.

#### 3.2.1. CFAM Model of the Single-Phase Bridge Rectifier Under Intermittent Current Conditions

Considering the impact of harmonics, the voltage at PCC can be expressed as $v_{\mathrm{ac}}\left(t\right)=\sum_{k=1}^{K}\sqrt{2}V_{k}\sin\left(k\omega t+\varphi_{k}\right)$, where *k* is an odd integer. Taking the phase of the fundamental voltage as a reference, when ωt=α, the thyristors $T_{1}$ and $T_{4}$ conduct. The current $i_{k}$ induced by $v_{k}$ satisfies the loop equation:

(15) $$\left\{\begin{array}{c} \sqrt{2}V_{k}\sin\left(k\omega t+\varphi_{k}\right)=L\frac{d}{dt}i_{k}\left(t\right)+Ri_{k}\left(t\right)\\ s.t.\;i_{k}\left(\alpha /\omega \right)=0 \end{array}\right.\;$$

By applying the Laplace transform, (15) can be transformed into the complex frequency domain for calculation. Subsequently, the time-domain expression of $i_{k}$ can be obtained through the inverse Laplace transform:

(16) $$i_{k}\left(t\right)=\frac{\sqrt{2}V_{k}}{\left|Z_{k}\right|}\left[\sin\left(k\omega t+\varphi \right)-A_{1}e^{\frac{R}{L}\left(\frac{\alpha }{\omega }-t\right)}\right]\;$$

where $Z_{k}=R+jk\omega L$, $\left|Z_{k}\right|$ and $\varphi_{Z}$ are the magnitude and phase of $Z_{k}$, respectively; $\varphi =\varphi_{k}-\varphi_{Z}$, $A_{1}=\sin\left(k\alpha +\varphi \right)$.

According to the piecewise linearization method, phase-controlled power electronic equipment behaves as a linear circuit within the individual operating stage and satisfies the superposition theorem. Consequently, the AC side current can be obtained by summing the currents induced by different order voltages in $v_{\mathrm{ac}}$, i.e., $i_{\mathrm{ac}}\left(t\right)=\sum_{k=1}^{K}i_{k}\left(t\right)$. The thyristor conduction angle is denoted as θ, and when ωt=α+θ, thyristors $T_{1}$ and $T_{4}$ are turned off. Combining (16) and substituting the condition $i_{\mathrm{ac}}\left(\left(\alpha +\theta \right)/\omega \right)=0$, we can obtain the expression for θ, denoted as $f\left(\theta \right)$, as shown in (17).

(17) $$f\left(\theta \right)=\sum_{k=1}^{K}\frac{\sqrt{2}V_{k}}{\left|Z_{k}\right|}\left[\sin\left(k\theta +k\alpha +\varphi \right)-A_{1}e^{-\frac{R\theta }{\omega L}}\right]\;$$

The value of θ can be calculated by setting f(θ)=0. Since the expression of f(θ) involves trigonometric and exponential functions, it belongs to the category of transcendental equations, making it difficult to derive an analytical solution directly. Consequently, this manuscript adopts the Newton–Raphson method to calculate the numerical solution of θ through iterative approximation. The specific solution steps are outlined in Algorithm 1.
**Algorithm 1** Newton–Raphson algorithm for calculating conduction angle1:**Input:**2:*f*, f(θ) for root finding3:$\theta_{0}$, initial guess of conduction angle4:ϵ, error tolerance5:*N*, max iterations6:**Output:** Conduction angle or error message.7:k←0, $\theta \leftarrow \theta_{0}$, e←ϵ+18:**while** (e>ϵ)∧(k<N) **do**9:      y←f(θ), $y^{\prime }\leftarrow f^{\prime }\left(\theta \right)$10:    **if** $|y^{\prime }|<10^{-10}$ **then return** “Error: Derivative near zero”11:    **end if**12:    $\theta \leftarrow \theta -y/y^{\prime }$, $e\leftarrow |\theta -\theta_{prev}|$, k←k+113:**end while**14:**if** e≤ϵ **then** **return** θ15:**elsereturn** “No convergence”16:**end if**

Based on the half-wave symmetry property of the odd harmonic function, the AC side current during the conduction of thyristors $T_{2}$ and $T_{3}$ can be obtained by translating and flipping the current in (16). Within a fundamental period (ωt∈[α,α+2π]), the AC side current $i_{k}^{\mathrm{ac}}$ induced by $v_{k}$ can be expressed as:

(18) $$i_{k}^{\mathrm{ac}}\left(t\right)=\left\{\begin{array}{cc} i_{k}\left(t\right), & \mathrm{if}\;\alpha <\omega t<\alpha +\theta \\ -i_{k}\left(t-\frac{\pi }{\omega }\right), & \mathrm{if}\;\alpha +\pi <\omega t<\alpha +\pi +\theta \\ 0, & \mathrm{otherwise}. \end{array}\right.\;$$

$i_{k}^{\mathrm{ac}}$ is a periodic function, which can be expanded into the trigonometric function form shown in (11) using Fourier series. After integration and trigonometric transformation, the result of $a_{h}$ is given by (19) and (20). Here, $a_{hh}$ and $a_{hk}$ represent the values of $a_{h}$ when h=k and h≠k, respectively; $C_{h}$ is the common term for $a_{hh}$ and $a_{hk}$, as expressed in (21); $Z_{h}=R+jh\omega L$, $\varphi_{Z_{h}}$ is the phase of $Z_{h}$ and *h* is odd. The form of $b_{h}$ is identical to $a_{h}$, with the only difference being that the sine functions in $a_{h}$ are replaced by cosine functions, and the cosine functions are replaced by sine functions. Due to space limitations, the specific expression of $b_{h}$ is not listed here. By substituting $a_{h}$ and $b_{h}$ into (14), the CFAM of the single-phase bridge rectifier under intermittent load current conditions can be calculated.

(19) $$a_{hh}=\frac{1}{2\pi k}\left[2k\theta \sin\varphi +\cos\left(2k\alpha +\varphi \right)-\cos\left(2k\alpha +2k\theta +\varphi \right)\right]+C_{h}\;$$

(20) $$\begin{array}{cc} a_{hk}= & \frac{\cos\left[\alpha \left(h+k\right)+\varphi \right]-\cos\left[\left(\alpha +\theta \right)\left(h+k\right)+\varphi \right]}{\pi \left(h+k\right)}\\ \; & -\frac{\cos\left[\alpha \left(h-k\right)-\varphi \right]-\cos\left[\left(\alpha +\theta \right)\left(h-k\right)-\varphi \right]}{\pi \left(h-k\right)}+C_{h} \end{array}\;$$

(21) $$C_{h}=\frac{2A_{1}\omega L}{\pi \left|Z_{h}\right|}\left[\cos\left(h\alpha +h\theta +\varphi_{Z_{h}}\right)e^{-\frac{R\theta }{\omega L}}-\cos\left(h\alpha +\varphi_{Z_{h}}\right)\right]$$

#### 3.2.2. CFAM Model of the Single-Phase Bridge Rectifier Under Continuous Current Conditions

Compared to the intermittent current condition, the initial current of the thyristors under continuous current conditions is no longer zero, and the thyristor conduction angle θ=π. When ωt=α, thyristors $T_{1}$ and $T_{4}$ conduct, and the DC side load is supplied by $v_{\mathrm{ac}}$. When ωt=α+π, thyristors $T_{1}$ and $T_{4}$ are turned off, another set of thyristors $T_{2}$ and $T_{3}$ conduct, and the DC side load is supplied by $-v_{\mathrm{ac}}$.

When thyristors $T_{1}$ and $T_{4}$ conduct, the current $i_{k}$ caused by the *k*-th voltage $v_{k}$ satisfies the equation shown in (22), where $I_{0}$ is the initial value of the current.

(22) $$\left\{\begin{array}{c} \sqrt{2}V_{k}\sin\left(k\omega t+\varphi_{k}\right)=L\frac{d}{dt}i_{k}\left(t\right)+Ri_{k}\left(t\right)\\ \begin{array}{cc} s.t. & i_{k}\left(\alpha /\omega \right)=I_{0}\\ \; & i_{k}\left(\left(\alpha +\pi \right)/\omega \right)=I_{0} \end{array} \end{array}\right.\;$$

By solving the differential equation, the analytical expressions for $I_{0}$ and $i_{k}$ can be derived, as given in (23) and (24). Here, $A_{2}=\frac{2A_{1}}{1-\exp\left(-\frac{\pi R}{\omega L}\right)}$.

(23) $$I_{0}=-\frac{A_{2}V_{k}}{\sqrt{2}\left|Z_{k}\right|}\left(1+e^{-\frac{\pi R}{\omega L}}\right)\;$$

(24) $$i_{k}\left(t\right)=\frac{\sqrt{2}V_{k}}{\left|Z_{k}\right|}\left[\sin\left(k\omega t+\varphi \right)-A_{2}e^{\frac{R}{L}\left(\frac{\alpha }{\omega }-t\right)}\right]\;$$

Utilizing the half-wave symmetry property, the expression of the AC side current caused by $v_{k}$ over one fundamental period can be expressed as:

(25) $$i_{k}^{\mathrm{ac}}\left(t\right)=\left\{\begin{array}{cc} i_{k}\left(t\right) & \mathrm{if}\;\alpha <\omega t<\alpha +\pi \\ -i_{k}\left(t-\frac{\pi }{\omega }\right) & \mathrm{if}\;\alpha +\pi <\omega t<\alpha +2\pi \end{array}\right.\;$$

The Fourier series decomposition of $i_{k}^{\mathrm{ac}}$ is carried out and expressed in the trigonometric form shown in (11). The Fourier coefficients $a_{hh}$ and $a_{hk}$ are given by (26) and (27). The coefficient $b_{h}$ exhibits the same characteristics as under the intermittent load current condition, and the specific expression is omitted here due to space constraints. By substituting the Fourier coefficients into (14), the CFAM of the single-phase bridge rectifier under continuous load current conditions can be obtained.

(26) $$a_{hh}=-\frac{2A_{2}\omega L}{\pi \left|Z_{h}\right|}\cos\left(h\alpha +\varphi_{Z_{h}}\right)\left(1+e^{-\frac{\pi R}{\omega L}}\right)+\sin\varphi \;$$

(27) $$a_{hk}=-\frac{2A_{2}\omega L}{\pi \left|Z_{h}\right|}\cos\left(h\alpha +\varphi_{Z_{h}}\right)\left(1+e^{-\frac{\pi R}{\omega L}}\right)$$

#### 3.2.3. Unified CFAM Model of the Single-Phase Bridge Rectifier

When the single-phase bridge rectifier operates under intermittent current conditions, the thyristor conduction angle $\theta \in \left(0,\pi \right)$; while under continuous current conditions, θ=π. Substituting θ=π into the Fourier coefficient expressions under intermittent current conditions ((19) and (20)), we find that the result is fully consistent with the form of (26) and (27). Consequently, the unified CFAM model of the single-phase bridge rectifier can adopt the Fourier coefficient expressions under intermittent load current conditions. This only requires replacing $A_{1}$ with *A* in (19) and (20), where *A* is defined in (28).

(28) $$A=\left\{\begin{array}{cc} \sin\left(k\alpha +\varphi_{k}-\varphi_{Z}\right), & \mathrm{if}\;0<\theta <\pi \\ \frac{2}{1-\exp\left(-\frac{\pi R}{\omega L}\right)}\sin\left(k\alpha +\varphi_{k}-\varphi_{Z}\right), & \mathrm{if}\;\theta =\pi \end{array}\right.\;$$

The peak DC voltage $V_{k\mathrm{dc}}$ of the single-phase bridge rectifier is equal to the peak AC voltage $V_{km}$ when the thyristors conduct. Based on (14), the CFAM element $Y_{hk}$ of the single-phase bridge rectifier can be calculated by:

(29) $$Y_{hk}=\left\{\begin{array}{cc} \frac{1}{\left|Z_{k}\right|}\left[a_{hh}∠\left(\frac{\pi }{2}-\varphi_{k}\right)+b_{hh}∠\left(-\varphi_{k}\right)\right], & \mathrm{if}\;h=k\\ \frac{1}{\left|Z_{k}\right|}\left[a_{hk}∠\left(\frac{\pi }{2}-\varphi_{k}\right)+b_{hk}∠\left(-\varphi_{k}\right)\right], & \mathrm{if}\;h\neq k \end{array}\right.\;$$

### 3.3. Construction of CFAM Analytical Model for the Three-Phase Bridge Rectifier

**Figure 5:**
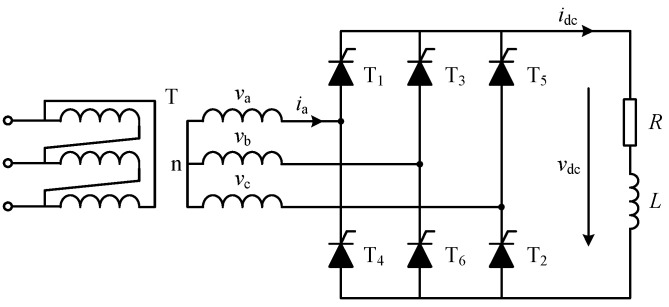
Three phase bridge-controlled rectifier circuit with resistive-inductive load.

The three-phase bridge rectifier is widely used in motor drive and speed control systems. In this section, the CFAM model of the three-phase bridge-controlled rectifier circuit shown in Figure 5 is developed using the proposed method.

**Figure 6:**
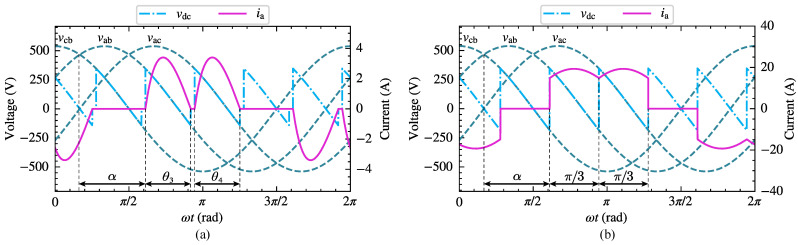
Schematic waveforms of line voltages, AC side phase-A current $i_{a}$, and DC side voltage $v_{\mathrm{dc}}$ of the three-phase bridge rectifier. (**a**) Intermittent current condition. (**b**) Continuous current condition.

Three phase bridge-controlled rectifiers are commonly used to supply inductive-resistive loads, and the operating characteristics are affected by both the load and thyristor trigger angle α. When α is less than $60^{\circ }$, the load current remains continuous. However, when α exceeds $60^{\circ }$, the continuity of the load current depends on the loop impedance angle. Figure 6 illustrates the voltage and current waveforms of the three-phase bridge-controlled rectifier circuit under intermittent and continuous load current conditions. In the following analysis, the CFAM analytical models for both operating conditions will be constructed using the proposed method.

#### 3.3.1. CFAM Model of the Three-Phase Bridge Rectifier Under Intermittent Current Conditions

The three-phase bridge-controlled rectifier circuit consists of six thyristors, which can be divided into a common cathode group ($T_{1},T_{3},T_{5}$) and a common anode group ($T_{4},T_{6},T_{2}$) in accordance with the different connection modes. During normal operation, the trigger circuit generates appropriate pulse signals to control the conduction sequence and timing of the thyristors in both groups, thus regulating the rectified output voltage. As shown in Figure 6a, when the load current is intermittent, the phase-A current on the AC side exhibits two non-zero operating stages within the positive half fundamental period, corresponding to thyristor conduction angles $\theta_{3}$ and $\theta_{4}$, respectively.

With the phase of phase-A fundamental voltage at PCC as reference, when $\omega t\in \left[\alpha +\frac{\pi }{6},\alpha +\frac{\pi }{6}+\theta_{3}\right]$, thyristors $T_{1}$, $T_{6}$ conduct and the load is supplied by the line voltage $v_{\mathrm{ab}}$. Considering the effects of harmonic voltages, the phase-A voltage at PCC can be expressed as $v_{a}\left(t\right)=\sum_{k=1}^{K}\sqrt{2}V_{k}\sin\left(k\omega t+\varphi_{k}\right)$, where *k* is odd. When thyristors $T_{1}$ and $T_{6}$ conduct, the current caused by the *k*-th voltage $v_{k}$ satisfies the circuit equation:

(30) $$\left\{\begin{array}{c} \sqrt{6}V_{k}\sin\left(k\omega t+\varphi_{k}+\frac{\pi }{6}\right)=Ri_{k1}\left(t\right)+L\frac{d}{dt}i_{k1}\left(t\right)\\ s.t.\;\;i_{k1}\left(\frac{\pi /6+\alpha }{\omega }\right)=0 \end{array}\right.\;$$

Using the Laplace transform to solve the first-order linear differential equation, the expression of the current $i_{k1}$ can be derived as:

(31) $$i_{k1}\left(t\right)=\frac{\sqrt{6}V_{k}}{\left|Z_{k}\right|}\left[\sin\left(k\omega t+\varphi_{3}\right)-A_{3}e^{\frac{R}{L}\left(\frac{\pi /6+\alpha }{\omega }-t\right)}\right]\;$$

where $\varphi_{3}=\varphi_{k}+\frac{\pi }{6}-\varphi_{Z}$, $A_{3}=\sin\left(k\alpha +\frac{k\pi }{6}+\varphi_{3}\right)$.

When thyristors $T_{1}$ and $T_{6}$ conduct, the phase-A current on the AC side is expressed in (32). Thyristors $T_{1}$ and $T_{6}$ are turned off at $\omega t=\alpha +\frac{\pi }{6}+\theta_{3}$, causing the loop current becomes zero. By combining (31) and (32) and following the solving steps in Algorithm 1, the value of $\theta_{3}$ can be determined.

(32) $$i_{a}\left(t\right)=\sum_{k=1}^{K}i_{k1}\left(t\right)\;$$

When $\omega t\in \left[\alpha +\frac{\pi }{2},\alpha +\frac{\pi }{2}+\theta_{4}\right]$, thyristors $T_{1}$ and $T_{2}$ conduct, and the load is supplied by the line voltage $v_{\mathrm{ac}}$. During this period, the current $i_{k2}$ induced by $v_{k}$ satisfies the circuit equation:

(33) $$\left\{\begin{array}{c} \sqrt{6}V_{k}\sin\left(k\omega t+\varphi_{k}-\frac{\pi }{6}\right)=Ri_{k2}\left(t\right)+L\frac{d}{dt}i_{k2}\left(t\right)\\ s.t.\;\;i_{k2}\left(\frac{\pi /2+\alpha }{\omega }\right)=0 \end{array}\right.$$

By solving the differential equation, the analytical expression of $i_{k2}$ can be obtained, as shown in (34). Here, $\varphi_{4}=\varphi_{k}-\frac{\pi }{6}-\varphi_{Z}$, $A_{4}=\sin\left(k\alpha +\frac{k\pi }{2}+\varphi_{4}\right)$.

(34) $$i_{k2}\left(t\right)=\frac{\sqrt{6}V_{k}}{\left|Z_{k}\right|}\left[\sin\left(k\omega t+\varphi_{4}\right)-A_{4}e^{\frac{R}{L}\left(\frac{\pi /2+\alpha }{\omega }-t\right)}\right]$$

The calculation method of $\theta_{4}$ is the same as that of $\theta_{3}$ and is not elaborated here. According to the half-wave symmetry of the odd harmonic function, the analytical expression of the phase-A current on the AC side induced by $v_{k}$ within one fundamental period ($\omega t\in [\alpha +\frac{\pi }{6},\alpha +\frac{13\pi }{6}]$) is given in (35).

(35) $$i_{k}^{a}\left(t\right)=\left\{\begin{array}{cc} i_{k1}\left(t\right), & \mathrm{if}\;\alpha +\frac{\pi }{6}<\omega t<\alpha +\theta_{3}+\frac{\pi }{6}\\ i_{k2}\left(t\right), & \mathrm{if}\;\alpha +\frac{\pi }{2}<\omega t<\alpha +\theta_{4}+\frac{\pi }{2}\\ -i_{k1}\left(t-\frac{\pi }{\omega }\right), & \mathrm{if}\;\alpha +\frac{7\pi }{6}<\omega t<\alpha +\theta_{3}+\frac{7\pi }{6}\\ -i_{k2}\left(t-\frac{\pi }{\omega }\right), & \mathrm{if}\;\alpha +\frac{3\pi }{2}<\omega t<\alpha +\theta_{4}+\frac{3\pi }{2}\\ 0, & \mathrm{otherwise}. \end{array}\right.\;$$

Using Fourier series, $i_{k}^{a}$ is expanded into the trigonometric function form shown in (11). After integration and trigonometric transformations, the expressions of $a_{hh}$ and $a_{hk}$ are, respectively, given in (36) and (37), with the common term $C_{h}$ provided in (38). The form of $b_{h}$ is identical to that of $a_{h}$, with the only difference being that the sine functions in $a_{h}$ are replaced by cosine functions, and the cosine functions are replaced by sine functions. Due to space limitations, the specific expression of $b_{h}$ is not listed here. By substituting $a_{h}$ and $b_{h}$ into (14), the CFAM of the three-phase bridge rectifier under intermittent load current conditions can be obtained.

(36) $$\begin{array}{cc} a_{hh}= & \frac{1}{2\pi k}\left[2k\theta_{3}\sin\varphi_{3}+2k\theta_{4}\sin\varphi_{4}+\cos\left(2k\alpha +\frac{\pi k}{3}+\varphi_{3}\right)-\cos\left(2k\alpha +\varphi_{4}\right)\right.\\ \; & \left.+\cos\left(2k\alpha +2k\theta_{4}+\varphi_{4}\right)-\cos\left(2k\alpha +2k\theta_{3}+\frac{\pi k}{3}+\varphi_{3}\right)\right]+C_{h} \end{array}\;$$

(37) $$\begin{array}{cc} a_{hk}= & \frac{1}{\pi \left(h+k\right)}\left\{\cos\left[\left(h+k\right)\left(\alpha +\frac{\pi }{6}\right)+\varphi_{3}\right]-\cos\left[\left(h+k\right)\left(\alpha +\theta_{3}+\frac{\pi }{6}\right)+\varphi_{3}\right]\right.\\ \; & \left.+\cos\left[\left(h+k\right)\left(\alpha +\frac{\pi }{2}\right)+\varphi_{4}\right]-\cos\left[\left(h+k\right)\left(\alpha +\theta_{4}+\frac{\pi }{2}\right)+\varphi_{4}\right]\right\}\\ \; & -\frac{1}{\pi \left(h-k\right)}\left\{\cos\left[\left(h-k\right)\left(\alpha +\frac{\pi }{6}\right)-\varphi_{3}\right]-\cos\left[\left(h-k\right)\left(\alpha +\theta_{3}+\frac{\pi }{6}\right)-\varphi_{3}\right]\right.\\ \; & \left.+\cos\left[\left(h-k\right)\left(\alpha +\frac{\pi }{2}\right)-\varphi_{4}\right]-\cos\left[\left(h-k\right)\left(\alpha +\theta_{4}+\frac{\pi }{2}\right)-\varphi_{4}\right]\right\}+C_{h} \end{array}$$

(38) $$\begin{array}{cc} C_{h}= & \frac{2A_{3}\omega L}{\pi \left|Z_{h}\right|}\left\{\cos\left[h\left(\alpha +\theta_{3}+\frac{\pi }{6}\right)+\varphi_{Z_{h}}\right]e^{-\frac{R\theta_{3}}{\omega L}}-\cos\left[h\left(\alpha +\frac{\pi }{6}\right)+\varphi_{Z_{h}}\right]\right\}\\ \; & +\frac{2A_{4}\omega L}{\pi \left|Z_{h}\right|}\left\{\cos\left[h\left(\alpha +\theta_{4}+\frac{\pi }{2}\right)+\varphi_{Z_{h}}\right]e^{-\frac{R\theta_{4}}{\omega L}}-\cos\left[h\left(\alpha +\frac{\pi }{2}\right)+\varphi_{Z_{h}}\right]\right\} \end{array}$$

#### 3.3.2. CFAM Model of the Three-Phase Bridge Rectifier Under Continuous Current Conditions

As depicted in Figure 6b, when the load current is continuous, the phase-A current on the AC side also exhibits two non-zero operating stages within the positive half fundamental period, and the corresponding thyristor conduction angles are both $\frac{\pi }{3}$.

When $\omega t\in [\alpha +\frac{\pi }{6},\alpha +\frac{\pi }{2}]$, thyristors $T_{1}$ and $T_{6}$ conduct, and the load is supplied by the line voltage $v_{\mathrm{ab}}$. The current $i_{k1}$ induced by the *k*-th voltage $v_{k}$ satisfies the circuit equation given in (39), where $I_{0}$ is the initial current.

(39) $$\left\{\begin{array}{c} \sqrt{6}V_{k}\sin\left(k\omega t+\varphi_{k}+\frac{\pi }{6}\right)=Ri_{k1}\left(t\right)+L\frac{d}{dt}i_{k1}\left(t\right)\\ s.t.\;\;i_{k1}\left(\frac{\pi /6+\alpha }{\omega }\right)=I_{0} \end{array}\right.\;$$

By applying the Laplace transform, the analytical expression of the current $i_{k1}$ can be calculated, as shown in (40). Here, $A_{5}=A_{3}-\frac{\left|Z_{k}\right|}{\sqrt{6}V_{k}}I_{0}$.

(40) $$i_{k1}\left(t\right)=\frac{\sqrt{6}V_{k}}{\left|Z_{k}\right|}\left[\sin\left(k\omega t+\varphi_{3}\right)-A_{5}e^{\frac{R}{L}\left(\frac{\pi /6+\alpha }{\omega }-t\right)}\right]\;$$

Based on $i_{k1}\left(\frac{\pi /2+\alpha }{\omega }\right)=I_{0}$, and combining with (40), the expression of $I_{0}$ can be be derived as:

(41) $$I_{0}=\frac{\sqrt{6}V_{k}}{\left|Z_{k}\right|}\frac{\sin\left(k\alpha +\frac{k\pi }{2}+\varphi_{3}\right)-\sin\left(k\alpha +\frac{k\pi }{6}+\varphi_{3}\right)e^{-\frac{\pi R}{3\omega L}}}{1-e^{-\frac{\pi R}{3\omega L}}}$$

When $\omega t\in \left[\alpha +\frac{\pi }{2},\alpha +\frac{5\pi }{6}\right]$, thyristor $T_{6}$ is off and $T_{2}$ conducts. The load is supplied by the line voltage $v_{\mathrm{ac}}$, and the current $i_{k2}$ induced by $v_{k}$ satisfies the circuit equation:

(42) $$\left\{\begin{array}{c} \sqrt{6}V_{k}\sin\left(k\omega t+\varphi_{k}-\frac{\pi }{6}\right)=Ri_{k2}\left(t\right)+L\frac{d}{dt}i_{k2}\left(t\right)\\ s.t.\;\;i_{k2}\left(\frac{\pi /2+\alpha }{\omega }\right)=I_{0} \end{array}\right.\;$$

The analytic expression of the current $i_{k2}$ is illustrated in (43), where $A_{6}=A_{4}-\frac{\left|Z_{k}\right|}{\sqrt{6}V_{k}}I_{0}$.

(43) $$i_{k2}\left(t\right)=\frac{\sqrt{6}V_{k}}{\left|Z_{k}\right|}\left[\sin\left(k\omega t+\varphi_{4}\right)-A_{6}e^{\frac{R}{L}\left(\frac{\pi /2+\alpha }{\omega }-t\right)}\right]\;$$

The time-domain expression of the phase-A current on the AC side caused by $v_{k}$ in one fundamental period can be obtained by employing the half-wave symmetry property, as presented in (44).

(44) $$i_{k}^{a}\left(t\right)=\left\{\begin{array}{cc} i_{k1}\left(t\right), & \mathrm{if}\;\alpha +\frac{\pi }{6}<\omega t<\alpha +\frac{\pi }{2}\\ i_{k2}\left(t\right), & \mathrm{if}\;\alpha +\frac{\pi }{2}<\omega t<\alpha +\frac{5\pi }{6}\\ -i_{k1}\left(t-\frac{\pi }{\omega }\right), & \mathrm{if}\;\alpha +\frac{7\pi }{6}<\omega t<\alpha +\frac{3\pi }{2}\\ -i_{k2}\left(t-\frac{\pi }{\omega }\right), & \mathrm{if}\;\alpha +\frac{3\pi }{2}<\omega t<\alpha +\frac{11\pi }{6}\\ 0, & \mathrm{otherwise}. \end{array}\right.\;$$

Through Fourier series decomposition, $i_{k}^{a}\left(t\right)$ can also be expressed in the trigonometric form shown in (11). The Fourier series coefficients $a_{hh}$ and $a_{hk}$ are calculated, with the results provided in (45) and (46), and the common term $C_{h}$ is given in (47). The coefficient $b_{h}$ exhibits the same properties as in the intermittent current condition, and the specific expression is omitted due to space limitations. By substituting the Fourier coefficients into (14), the CFAM of the three-phase bridge rectifier can be calculated under continuous load current conditions.

(45) $$\begin{array}{cc} a_{hh}= & \frac{1}{2\pi k}\left[\frac{2\pi k}{3}\sin\varphi_{3}+\frac{2\pi k}{3}\sin\varphi_{4}+\cos\left(2k\alpha +\frac{\pi k}{3}+\varphi_{3}\right)-\cos\left(2k\alpha +\varphi_{4}\right)\right.\\ \; & \left.+\cos\left(2k\alpha +\frac{2\pi k}{3}+\varphi_{4}\right)+\cos\left(2k\alpha +\varphi_{3}\right)\right]+C_{h} \end{array}$$

(46) $$\begin{array}{cc} a_{hk}= & \frac{1}{\pi \left(h+k\right)}\left\{\cos\left[\left(h+k\right)\left(\alpha +\frac{\pi }{6}\right)+\varphi_{3}\right]-\cos\left[\left(h+k\right)\left(\alpha +\frac{\pi }{2}\right)+\varphi_{3}\right]\right.\\ \; & \left.+\cos\left[\left(h+k\right)\left(\alpha +\frac{\pi }{2}\right)+\varphi_{4}\right]-\cos\left[\left(h+k\right)\left(\alpha +\frac{5\pi }{6}\right)+\varphi_{4}\right]\right\}\\ \; & -\frac{1}{\pi \left(h-k\right)}\left\{\cos\left[\left(h-k\right)\left(\alpha +\frac{\pi }{6}\right)-\varphi_{3}\right]-\cos\left[\left(h-k\right)\left(\alpha +\frac{\pi }{2}\right)-\varphi_{3}\right]\right.\\ \; & \left.+\cos\left[\left(h-k\right)\left(\alpha +\frac{\pi }{2}\right)-\varphi_{4}\right]-\cos\left[\left(h-k\right)\left(\alpha +\frac{5\pi }{6}\right)-\varphi_{4}\right]\right\}+C_{h} \end{array}$$

(47) $$\begin{array}{cc} C_{h}= & \frac{2A_{5}\omega L}{\pi \left|Z_{h}\right|}\left[\cos\left(h\alpha +\frac{\pi h}{2}+\varphi_{Z_{h}}\right)e^{-\frac{\pi R}{3\omega L}}-\cos\left(h\alpha +\frac{\pi h}{6}+\varphi_{Z_{h}}\right)\right]\\ \; & +\frac{2A_{6}\omega L}{\pi \left|Z_{h}\right|}\left[\cos\left(h\alpha +\frac{5\pi h}{6}+\varphi_{Z_{h}}\right)e^{-\frac{\pi R}{3\omega L}}-\cos\left(h\alpha +\frac{\pi h}{2}+\varphi_{Z_{h}}\right)\right] \end{array}$$

#### 3.3.3. Unified CFAM Model of the Three-Phase Bridge Rectifier

In the case of the three-phase bridge rectifier under continuous current conditions, the thyristor conduction angle is $\frac{\pi }{3}$, whereas under intermittent current conditions, the thyristor conduction angle ranges between 0 and $\frac{\pi }{3}$. By substituting $\theta_{3}=\frac{\pi }{3}$ and $\theta_{4}=\frac{\pi }{3}$ into the Fourier coefficients expressions under intermittent current conditions ((36) and (37)), it is found that the result matches exactly the form of (45) and (46). As a result, the unified CFAM model of the three-phase bridge rectifier can use the Fourier coefficient expressions under intermittent current conditions. This only requires replacing $A_{3}$ and $A_{4}$ in (36) and (37) with $A_{\mathrm{III}}$ and $A_{\mathrm{IV}}$, respectively. The expressions for $A_{\mathrm{III}}$ and $A_{\mathrm{IV}}$ are given in (48) and (49).

(48) $$A_{\mathrm{III}}=\left\{\begin{array}{cc} \sin\left(k\alpha +\frac{k\pi }{6}+\varphi_{3}\right), & \mathrm{if}\;0<\theta <\frac{\pi }{3}\\ \sin\left(k\alpha +\frac{k\pi }{6}+\varphi_{3}\right)-\frac{\left|Z_{k}\right|}{\sqrt{6}V_{k}}I_{0}, & \mathrm{if}\;\theta =\frac{\pi }{3} \end{array}\right.$$

(49) $$A_{\mathrm{IV}}=\left\{\begin{array}{cc} \sin\left(k\alpha +\frac{k\pi }{2}+\varphi_{4}\right), & \mathrm{if}\;0<\theta <\frac{\pi }{3}\\ \sin\left(k\alpha +\frac{k\pi }{2}+\varphi_{4}\right)-\frac{\left|Z_{k}\right|}{\sqrt{6}V_{k}}I_{0}, & \mathrm{if}\;\theta =\frac{\pi }{3} \end{array}\right.$$

When the switching devices conduct, the DC side load of the three-phase bridge rectifier is supplied by the line voltage at PCC. Consequently, the peak voltage $V_{k\mathrm{dc}}$ of the DC side load is $\sqrt{3}$ times the peak voltage $V_{km}$ of phase-A at PCC. According to (14), the CFAM element $Y_{hk}$ of the three-phase bridge rectifier can be calculated by:

(50) $$Y_{hk}=\left\{\begin{array}{cc} \frac{\sqrt{3}}{\left|Z_{k}\right|}\left[a_{hh}∠\left(\frac{\pi }{2}-\varphi_{k}\right)+b_{hh}∠\left(-\varphi_{k}\right)\right], & \mathrm{if}\;h=k\\ \frac{\sqrt{3}}{\left|Z_{k}\right|}\left[a_{hk}∠\left(\frac{\pi }{2}-\varphi_{k}\right)+b_{hk}∠\left(-\varphi_{k}\right)\right], & \mathrm{if}\;h\neq k \end{array}\right.$$

## 4. Model Validation and Case Studies

In this section, the effectiveness and accuracy of the proposed harmonic source modeling method are verified through Matlab/Simulink simulations and experimental tests. First, extensive simulations are conducted under both intermittent and continuous load current conditions for the single-phase bridge rectifier circuit in typical harmonic scenarios. The CFAM analytical model, established using the proposed method, is employed for harmonic modeling and compared with the traditional CCS model for analysis. Next, numerous time-domain simulations are performed on the three-phase bridge rectifier circuit under varying circuit control parameters and power supply voltage distortions, which validates the effectiveness and precision advantages of the proposed method. Finally, an experimental platform for the three-phase bridge rectifier is set up, and experiments are conducted under different thyristor trigger angles and load inductances to further verify the validity of the proposed CFAM analytical model.

The harmonic source simulation models are developed on the MATLAB/Simulink R2022a simulation platform. During the simulation process, voltage and current waveforms at PCC are collected with a sampling frequency of 12.8 kHz. To account for the influence of random factors such as sampling errors, a maximum random error of ±1% is introduced into the voltage and current waveform data. Additionally, the time window for performing FFT analysis on the waveform data is set to 100 ms.

### 4.1. Evaluation Metrics for Harmonic Source Modeling

To quantitatively evaluate the accuracy of harmonic source models, the current amplitude mean relative error ${\bar{e}}_{\mathrm{IA}}^{h}$, the current phase mean absolute error ${\bar{e}}_{\mathrm{IP}}^{h}$, and the current waveform deviation coefficient $\varepsilon_{w}$ are introduced, as defined in (51)–(53).

(51) $${\bar{e}}_{\mathrm{IA}}^{h}\left(\%\right)=\frac{100}{N_{h}}\sum_{h=1}^{H}\left|\frac{I_{h}^{M}-I_{h}^{C}}{I_{h}^{M}}\right|\;$$

(52) $${\bar{e}}_{\mathrm{IP}}^{h}=\frac{1}{N_{h}}\sum_{h=1}^{H}\left|\phi_{h}^{M}-\phi_{h}^{C}\right|$$

(53) $$\varepsilon_{w}\left(\%\right)=\frac{100}{I_{1}}\sqrt{\frac{1}{N_{s}}\sum_{n=1}^{N_{s}}{\left[i\left(t_{n}\right)-\hat{i}\left(t_{n}\right)\right]}^{2}}$$

where *h* represents the harmonic current order, *H* denotes the highest harmonic order considered, and $N_{h}$ is the total number of harmonics considered; $I_{h}^{M}$ and $I_{h}^{C}$ represent the measured and calculated values of the harmonic current amplitude, respectively; $\phi_{h}^{M}$ and $\phi_{h}^{C}$ denote the measured and calculated values of the harmonic current phase, respectively; $I_{1}$ is the measured rms value of the fundamental current, and $N_{s}$ denotes the number of current waveform sampling points used to calculate $\varepsilon_{w}$; $i\left(t_{n}\right)$ and $\hat{i}\left(t_{n}\right)$ represent the current measurements and model calculations at the *n*-th sampling point, respectively.

### 4.2. Simulation Validation of Unified CFAM Model for the Single-Phase Bridge Rectifier

#### 4.2.1. Typical Harmonic Scenario Settings and Single-Phase Bridge Rectifier Simulation

This part establishes the time-domain simulation model of the single-phase bridge rectifier circuit shown in Figure 2a and verifies the CFAM analytical model derived in Section 3.2. When the single-phase bridge rectifier operates under intermittent current conditions, the equivalent load resistance *R* and inductance *L* on the DC side are set to 30 Ω and 20 mH, respectively. The target value of the thyristor trigger angle α is set to $30^{\circ }$. Considering the effect of noise, white noise from a normal distribution with a mean of 0 and a standard deviation of 1 is added to the target value of α during simulation. When operating under continuous current conditions, the equivalent resistance *R* is set to 5 Ω, while the other parameters remain unchanged.

**Figure 7:**
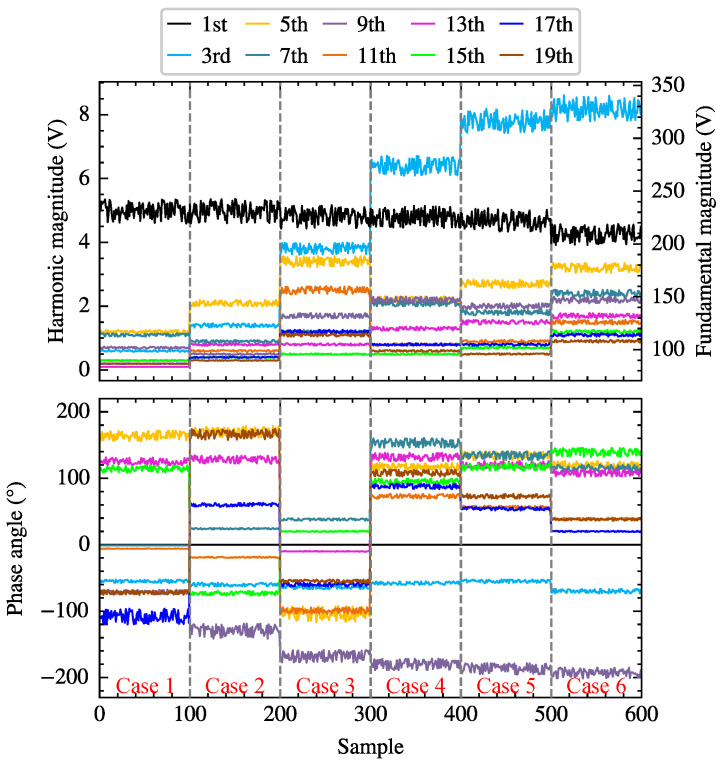
The magnitudes and phases of 600 supply voltages generated from the six typical harmonic scenarios.

Table 1 lists the measured PCC voltages under five typical harmonic scenarios (Case 1–Case 5) from [29], where the total harmonic distortion of the voltages $V_{\mathrm{THD}}$ ranges from 0.84% to 4.01%. Due to the relatively small variations in the fundamental voltage amplitude in Cases 1 to 5, with a maximum fluctuation of 3.50%, Case 6 is introduced on this basis, where the maximum fluctuation of the fundamental voltage in the cases reaches 10.60%. To simulate small fluctuations in PCC voltages, 100 different voltage combinations are generated from the five given scenarios separately according to (54). Figure 7 displays the magnitudes and phases of 600 supply voltages generated from the six harmonic scenarios. Simulations of the single-phase bridge rectifier circuit are conducted under various supply voltages and thyristor trigger angles. PCC voltages and currents are sampled, and FFT analysis is performed to extract the voltage and current measurements at different orders.

(54) $$u=u_{\mathrm{typ}}\times \left(0.95+0.1\times rand\right)\;$$

where *u* represents the magnitude and phase of the generated voltage at different orders; $u_{\mathrm{typ}}$ denotes the voltage magnitude or phase under the typical harmonic scenarios; and rand is a random number uniformly distributed within the range (0, 1).

#### 4.2.2. Verification of Unified CFAM Model for the Single-Phase Bridge Rectifier

Substituting PCC voltages and circuit parameters into (29), the CFAM parameters of the single-phase bridge rectifier are calculated. Based on the established CFAM analytical model, currents of different orders are further calculated, and the time-domain current waveforms are reconstructed. The simulation current under the voltage supply in Case 1 with $\alpha =30^{\circ }$ is used to establish the CCS model. Additionally, using the operating state with the voltage supply in Case 1 and $\alpha =30^{\circ }$ as the reference, the calculation process introduced in Section 2.1 is applied, and the CFAM model based on small signal theory is established according to (2) and (3). By substituting the model estimates and current measurements into (51)–(53), the evaluation metrics ${\bar{e}}_{\mathrm{IA}}^{h}$, ${\bar{e}}_{\mathrm{IP}}^{h}$, and $\varepsilon_{w}$ are calculated, respectively.

Table 2 and Table 3, respectively, list the mean and variance of the evaluation metrics for the harmonic models across 600 test samples under intermittent and continuous load current conditions. For convenience, in the following text, CFAM_pro represents the analytical CFAM model calculated using the proposed method, while CFAM_tra represents the CFAM model calculated based on the traditional small signal theory method. Under both operating conditions, the mean value of the current amplitude relative error $\mu_{{\bar{e}}_{\mathrm{IA}}^{h}}$ for the proposed CFAM model is less than 3%, and the mean value of the current phase absolute error $\mu_{{\bar{e}}_{\mathrm{IP}}^{h}}$ is less than $4^{\circ }$. The results demonstrate that the harmonic currents calculated using the proposed CFAM model are very close to the measured values, which verifies the effectiveness of the proposed method. In contrast, the CCS model exhibits lower modeling accuracy, as it does not account for the impact of PCC voltage variations on harmonic currents. The CFAM model based on small signal theory relies on the operating point of the harmonic source, and noticeable errors arise when the operating state deviates considerably from this point. As a result, its harmonic modeling accuracy is lower than that of the analytical CFAM model established by the proposed method. Furthermore, comparing the variances of the evaluation metrics reveals that the proposed CFAM model provides more stable harmonic modeling performance, while the harmonic modeling accuracy of the CCS model and traditional CFAM model exhibits greater fluctuation.

**Figure 8:**
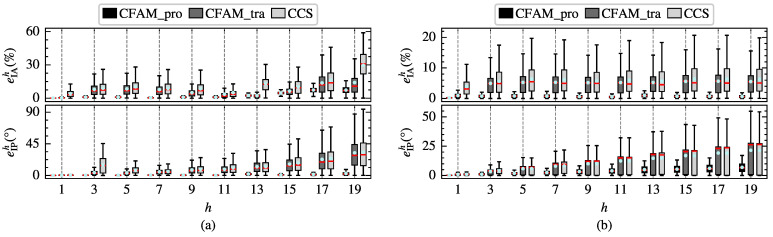
Distribution of the amplitude relative error $e_{\mathrm{IA}}^{h}$ and the phase absolute error $e_{\mathrm{IP}}^{h}$ of different order currents for the single-phase bridge rectifier. (**a**) Intermittent load current condition. (**b**) Continuous load current condition.

To further evaluate the modeling performance of the harmonic models, the amplitude relative error $e_{\mathrm{IA}}^{h}$ and the phase absolute error $e_{\mathrm{IP}}^{h}$ of different order currents are calculated. The box plots in Figure 8 illustrate the distribution of $e_{\mathrm{IA}}^{h}$ and $e_{\mathrm{IP}}^{h}$ for the proposed CFAM model, the CCS model and the traditional CFAM model. In particular, the red horizontal line represents the median of the errors, the upper and lower edges of the rectangular box correspond to the upper and lower quartiles of the errors, and the circular marker indicates the mean values of the errors. Under both operating conditions, the box plots for the CCS model and traditional CFAM model are wider than those for the CFAM model, indicating a larger fluctuation range of the errors. Furthermore, when comparing the error distributions across different order currents, it can be evident that the mean errors of the CFAM model are generally smaller than those of the CCS model and traditional CFAM model.

**Figure 9:**
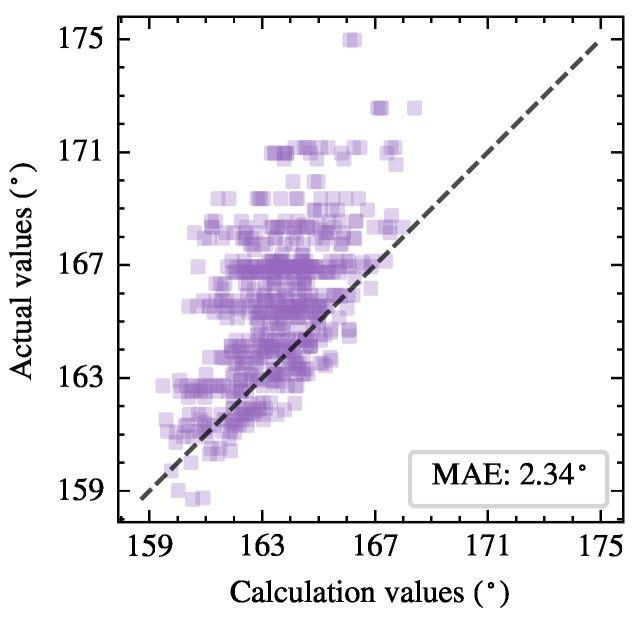
Distribution of calculated and simulated conduction angles of thyristors in 600 test samples.

The initial value of the thyristor conduction angle is set to π, and then the Newton–Raphson method in Algorithm 1 is employed for iterative calculation. Additionally, the simulation results of thyristor conduction angles can be extracted in batches from the simulated waveforms through programming. Figure 9 visually demonstrates the distribution of calculated and simulated values of the thyristor conduction angles. The closer the square markers are to the diagonal line, the more accurate the calculated results. The mean absolute error (MAE) of the conduction angles among the 600 test samples is $2.34^{\circ }$, which verifies the effectiveness and accuracy of the proposed calculation method.

**Figure 10:**
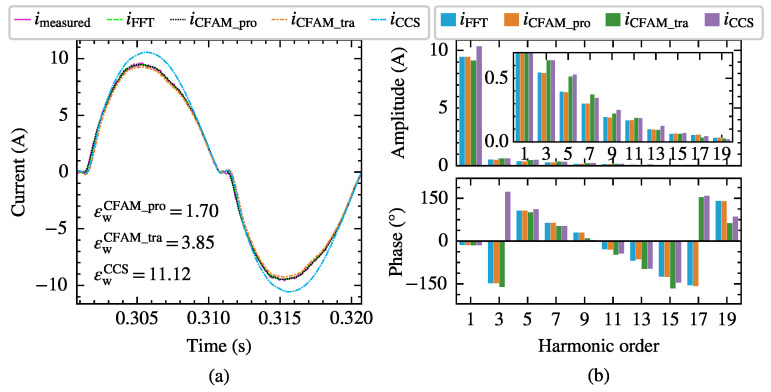
Comparison of the current measurements and model estimates for the single-phase bridge rectifier under intermittent current conditions when supplied by the voltage in Case 6. (**a**) Comparison of current waveforms. (**b**) Comparison of current amplitude spectrums and phase spectrums.

**Figure 11:**
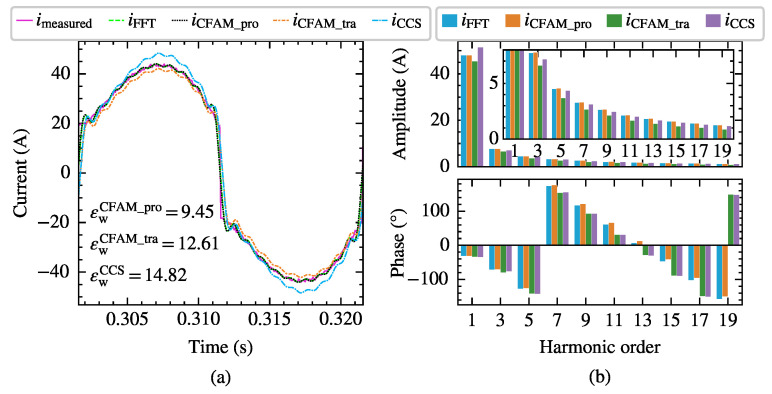
Comparison of the current measurements and model estimates for the single-phase bridge rectifier under continuous current conditions when supplied by the voltage in Case 6. (**a**) Comparison of current waveforms. (**b**) Comparison of current amplitude spectrums and phase spectrums.

Figure 10 and Figure 11, respectively, present the current measurements and model estimates for the single-phase bridge rectifier under intermittent and continuous load current conditions when supplied by the voltage in Case 6 with a trigger angle $\alpha =27^{\circ }$. In particular, $i_{\mathrm{measured}}$ represents the current waveform measured at PCC, $i_{\mathrm{FFT}}$ denotes the current waveform reconstructed from the first 19 odd harmonics of the measured current. Furthermore, $i_{\mathrm{CFAM}\_\mathrm{pro}}$, $i_{\mathrm{CFAM}\_\mathrm{tra}}$ and $i_{\mathrm{CCS}}$ indicate the current waveforms reconstructed from the current estimates using the proposed CFAM model, the traditional CFAM model and, and the CCS model, respectively. Consequently, the waveform difference between $i_{\mathrm{FFT}}$ and $i_{\mathrm{measured}}$ primarily reflects the harmonic current content above the 19th order. The current waveform deviation coefficient $\varepsilon_{w}$ marked in Figure 10a and Figure 11a represents the waveform difference between the estimated current and PCC measured current. By comparing the time-domain waveforms and frequency spectra, it can be observed that $i_{\mathrm{CFAM}\_\mathrm{pro}}$ closely matches $i_{\mathrm{FFT}}$, which further validates the effectiveness and accuracy of the proposed CFAM model.

**Figure 12:**
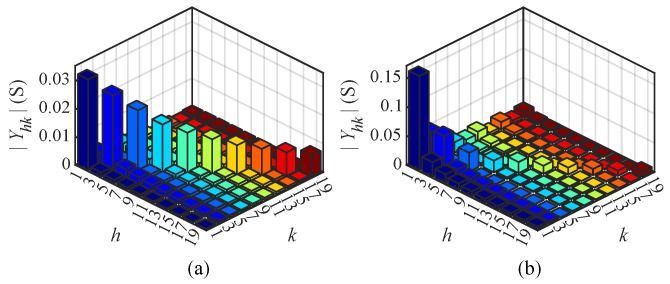
Magnitude $\left|Y_{hk}\right|$ of CFAM elements for the single-phase bridge rectifier under intermittent (**a**) and continuous (**b**) load current conditions in Case 6.

Figure 12 illustrates the magnitude $\left|Y_{hk}\right|$ of the CFAM elements under intermittent and continuous current conditions in Case 6, visually demonstrating the coupling relationship between the *h*-th current and *k*-th voltage. Analysis of different order currents reveals that the diagonal elements of the CFAM are larger than the non-diagonal elements under both operating conditions. Consequently, there is a strong coupling relationship between the harmonic current and the same-order voltage in the single-phase bridge rectifier.

### 4.3. Simulation Verification of Unified CFAM Model for the Three-Phase Bridge Rectifier

#### 4.3.1. Variable Parameter Simulation and Data Acquisition of the Three-Phase Bridge Rectifier

To verify the validity of the unified CFAM model for the three-phase bridge rectifier developed in Section 3.3, this part conducts a simulation analysis of the rectifier circuit shown in Figure 5. Under intermittent load current conditions, the equivalent resistance *R* and inductance *L* of the DC−side load is set to 50 Ω and 30 mH, respectively. Considering the influence of noise, the thyristor trigger angle α follows a normal distribution with a mean of $80^{\circ }$ and a standard deviation of $1^{\circ }$. While under continuous load current conditions, the load equivalent resistance *R* is set to 5 Ω, and the remaining parameters are the same as in the intermittent load current conditions. For the harmonic analysis of the three-phase bridge rectifier, only the characteristic harmonics up to the 25th order $\left(h=6k\pm 1,k=1,2,3,4\right)$ are considered.

**Figure 13:**
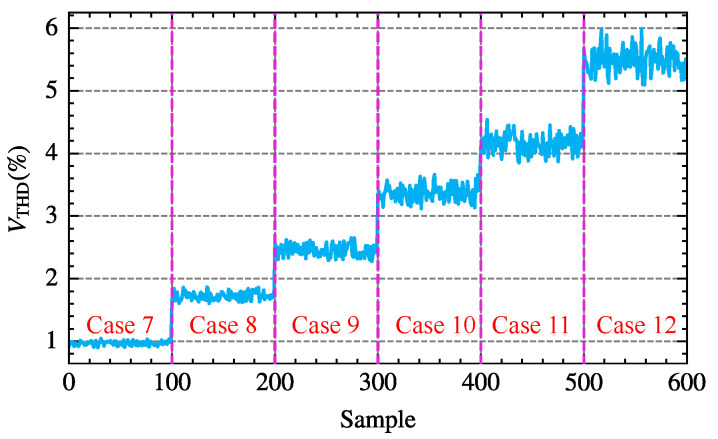
Distribution of total harmonic distortion $V_{\mathrm{THD}}$ across 600 different power voltage scenarios.

Table 4 lists six voltage cases with $V_{\mathrm{THD}}$ fluctuations ranging from 0.97% to 5.51%. For each given case, the voltage magnitude and phase at different orders are set with a certain range of fluctuations according to (54), generating 600 different supply voltage scenarios. Figure 13 illustrates the total harmonic distortion of the voltage $V_{\mathrm{THD}}$ for these scenarios. Simulations with varying parameters are conducted on the three-phase bridge rectifier circuit using the above supply voltages and thyristor trigger angles, and the voltage and current data of phase-A at PCC are sampled. Subsequently, waveform data from five fundamental periods are selected for FFT analysis to calculate the voltages and currents of different orders.

#### 4.3.2. Validation of Unified CFAM Model for the Three-Phase Bridge Rectifier

The CFAM model of the three-phase bridge rectifier is established by substituting the PCC voltages and circuit parameters into (50). The CCS model is calculated using the simulated current under the supplied voltage in Case 7 with $\alpha =80^{\circ }$. In addition, utilizing the operating state with the power supply voltage specified in Case 7 and $\alpha =80^{\circ }$ as the reference, the traditional CFAM model based on small signal theory is developed following the calculation procedure outlined in Section 2.1. The evaluation metrics ${\bar{e}}_{\mathrm{IA}}^{h}$, ${\bar{e}}_{\mathrm{IP}}^{h}$, and $\varepsilon_{w}$ are then calculated for 600 test samples based on the current measurements and the model estimates, respectively. Table 5 and Table 6 list the mean and variance of the evaluation metrics for the harmonic models under intermittent and continuous load current conditions. The results indicate that the mean value of the current amplitude relative error $\mu_{{\bar{e}}_{\mathrm{IA}}^{h}}$ for the proposed CFAM model is less than 5.5%, and the mean value of the current phase absolute error $\mu_{{\bar{e}}_{\mathrm{IP}}^{h}}$ is less than $6^{\circ }$. Furthermore, comparing the mean and variance of the evaluation metrics, it can be observed that the proposed CFAM model exhibits superior accuracy and stability in harmonic modeling than the CCS model and traditional CFAM model under both operating conditions.

**Figure 14:**
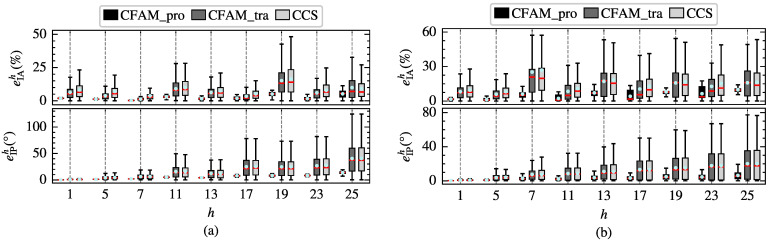
Distribution of the amplitude relative error $e_{\mathrm{IA}}^{h}$ and the phase absolute error $e_{\mathrm{IP}}^{h}$ of different order currents for the three-phase bridge rectifier. (**a**) Intermittent load current condition. (**b**) Continuous load current condition.

To further investigate the harmonic modeling performance of the harmonic models for the three-phase bridge rectifier, the amplitude relative error $e_{\mathrm{IA}}^{h}$ and the phase absolute error $e_{\mathrm{IP}}^{h}$ of different order currents in the test samples are calculated. Figure 14 displays the distribution of $e_{\mathrm{IA}}^{h}$ and $e_{\mathrm{IP}}^{h}$ for the three harmonic models under intermittent and continuous load current conditions. The results indicate that the mean errors and the box plot widths of the proposed CFAM model are generally smaller than those of the CCS model and traditional CFAM model, which further validates the superior accuracy of the proposed CFAM model in harmonic modeling.

**Figure 15:**
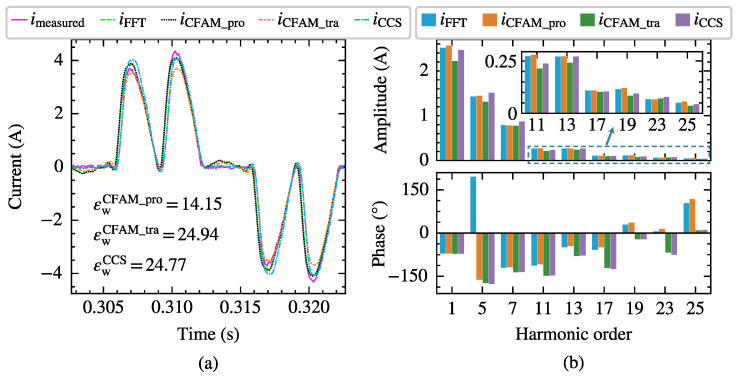
The current measurements and model estimates for the three-phase bridge rectifier under intermittent load current conditions when supplied by the voltage in Case 12. (**a**) Comparison of current waveforms. (**b**) Comparison of current amplitude spectrums and phase spectrums.

**Figure 16:**
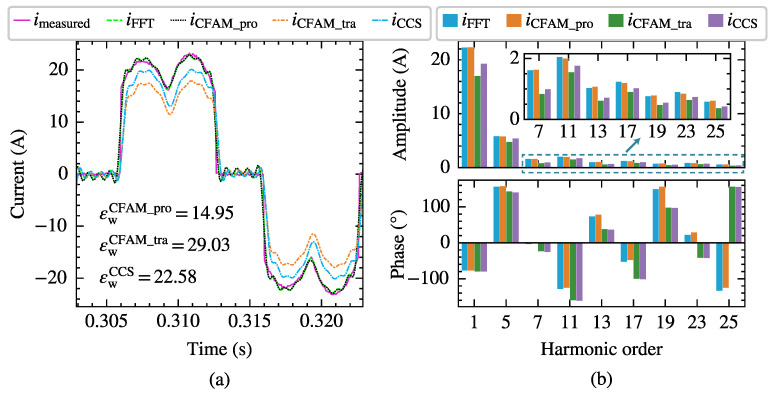
The current measurements and model estimates for the three-phase bridge rectifier under continuous load current conditions when supplied by the voltage in Case 12. (**a**) Comparison of current waveforms. (**b**) Comparison of current amplitude spectrums and phase spectrums.

Taking the scenario supplied by the voltage in Case 12 with a trigger angle $\alpha =77^{\circ }$ as an example, the harmonic modeling performance of the harmonic models for the three-phase bridge rectifier is compared. Figure 15 and Figure 16 present the waveforms and spectrum comparisons of the measured current and the model estimates under intermittent and continuous load current conditions, respectively.

In Figure 15a and Figure 16a, $i_{\mathrm{measured}}$ represents the current waveform measured at PCC, and $i_{\mathrm{FFT}}$ indicates the reconstructed current waveform derived from the fundamental and the first 25th-order characteristic harmonics from the measured current. Consequently, the waveform difference between $i_{\mathrm{measured}}$ and $i_{\mathrm{FFT}}$ mainly reflects the harmonic components above the 25th order. The current waveform deviation coefficient $\varepsilon_{w}$ annotated in the figures quantifies the waveform difference between the estimated current and measured current. When reconstructing the time-domain current waveform using harmonic models, only the fundamental and the first 25th-order characteristic harmonics are considered, resulting in a relatively large waveform difference compared to the measured current. From both the waveform and spectrum diagrams, it can be observed that the current $i_{\mathrm{CFAM}\_\mathrm{pro}}$ calculated using the proposed CFAM model is closer to the FFT analysis result $i_{\mathrm{FFT}}$, while the current $i_{\mathrm{CFAM}\_\mathrm{tra}}$ calculated by the traditional CFAM model and the current $i_{\mathrm{CCS}}$ calculated by the CCS model exhibits larger discrepancies. Furthermore, as evident from Figure 15 and Figure 16, the estimation errors of the traditional CFAM model for currents are significantly higher than the mean values of the corresponding error evaluation metrics presented in Table 5 and Table 6. In Case 12, the fundamental voltage decreases by 10.38% compared to Case 7, indicating that the CFAM model based on small signal theory relies on the operating point of the harmonic source and incurs noticeable errors when the operating state deviates from this point.

**Figure 17:**
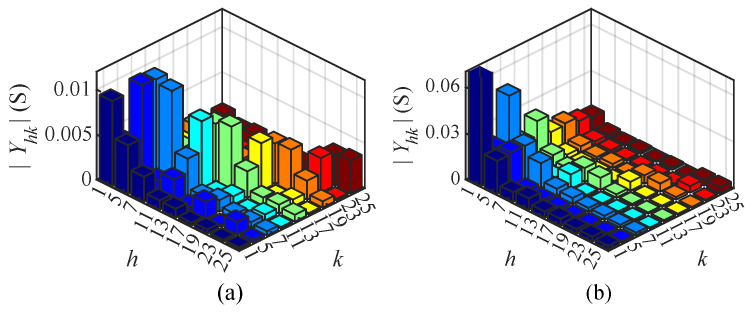
Magnitude $\left|Y_{hk}\right|$ of CFAM elements for the three-phase bridge rectifier under intermittent (**a**) and continuous (**b**) load current conditions in Case 12.

Figure 17 illustrates the magnitude $\left|Y_{hk}\right|$ of the CFAM elements under intermittent and continuous load current conditions in Case 12. By analyzing the currents of different orders, it can be found that the diagonal and some non-diagonal elements of the CFAM exhibit relatively large magnitudes in both operating conditions. This suggests that the harmonic current not only has a significant coupling relationship with the same-order voltage but also demonstrates a relatively strong coupling with some non-identical order voltages in the three-phase bridge rectifier.

### 4.4. Experimental Verification of the CFAM Analytical Model

**Figure 18:**
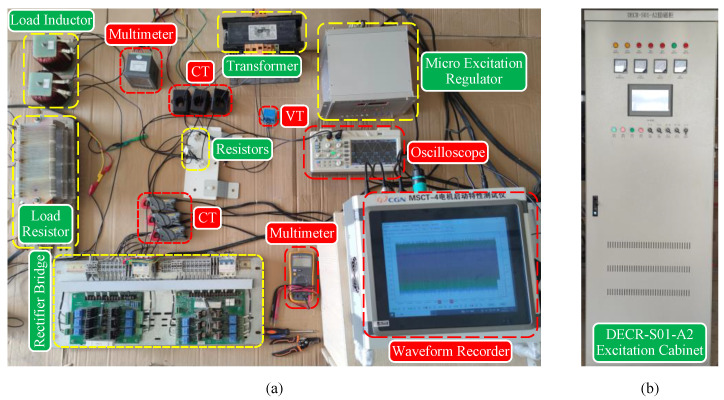
(**a**) Experimental test platform for the three-phase bridge rectifier. (**b**) Excitation cabinet.

Taking the three-phase bridge rectifier as an example, this part establishes an experimental test platform for the circuit shown in Figure 5 to verify the effectiveness of the proposed CFAM analytical model. Figure 18 presents the experimental test platform. The three-phase bridge rectifier used in the experiment is sourced from the excitation cabinet shown in Figure 18b. The thyristor trigger angle can be set through the microcomputer excitation regulator in Figure 18a, which in turn controls the output voltage of the rectifier. Due to constraints from the transformer capacity and the safety clearance of the thyristors in the rectifier, the output line voltage on the secondary winding of the transformer is set to 40 V. The measured values of the AC−side resistance and DC−side load resistance are 0.6 Ω and 5 Ω, respectively. The operating state of the rectifier is adjusted by varying the thyristor trigger angle and the load inductance during the experiment. A dual-trace oscilloscope is utilized to simultaneously sample the voltage across the AC−side resistance and the line voltage of the secondary winding, which can be further processed to obtain the current and voltage waveform data of phase A on the AC side. Table 7 and Table 8 list the circuit parameter settings and corresponding output voltage measurements of the rectifier under intermittent and continuous load current conditions, respectively. Here, α represents the thyristor trigger angle, *L* denotes the load inductance, $V_{\mathrm{dc}}$ is the measured output voltage of the three-phase rectifier on the DC side, and $S_{i}$ refers to the *i*-th operating state.

We performed FFT analysis on the collected waveform data to calculate different order voltages and currents under various operating states. Using the fundamental voltage phase as a reference, phase normalization was applied to each order voltage and current. It is worth noting that the harmonic phases need to be multiplied by the harmonic order when normalizing. The voltages and circuit parameters were then substituted into (50) to calculate the parameters of the CFAM analytical model for the three-phase bridge rectifier. Under the intermittent load current conditions, the CCS model and the CFAM model based on small signal theory were developed for load inductances of 1mH and 3mH, respectively, with $\alpha =80^{\circ }$ as the reference. Additionally, the CCS model and the traditional CFAM model were established under the continuous current conditions for load inductances of 1mH and 3mH, respectively, with $\alpha =50^{\circ }$ as the reference. Using different harmonic source models, the harmonic currents were calculated for each operating state. Finally, the evaluation metrics ${\bar{e}}_{\mathrm{IA}}^{h}$, ${\bar{e}}_{\mathrm{IP}}^{h}$, and $\varepsilon_{w}$ were computed based on the current measurements and the model estimates, respectively.

Table 9 and Table 10 list the mean and variance of the evaluation metrics for the three harmonic models across 20 test samples under intermittent and continuous load current conditions, respectively. Only the characteristic harmonics within the 25th order are considered during the calculation process $\left(h=6k\pm 1,k=1,2,3,4\right)$. The results illustrate that the mean value of the current amplitude relative error $\mu_{{\bar{e}}_{\mathrm{IA}}^{h}}$ for the proposed CFAM model is lower than 7.6%, and the mean value of the current phase absolute error $\mu_{{\bar{e}}_{\mathrm{IP}}^{h}}$ is lower than $10^{\circ }$. In addition, the proposed analytical CFAM model exhibits smaller means and variances for the evaluation metrics compared to the traditional CFAM model and CCS model, indicating higher estimation accuracy and more stable performance in harmonic current estimation.

**Figure 19:**
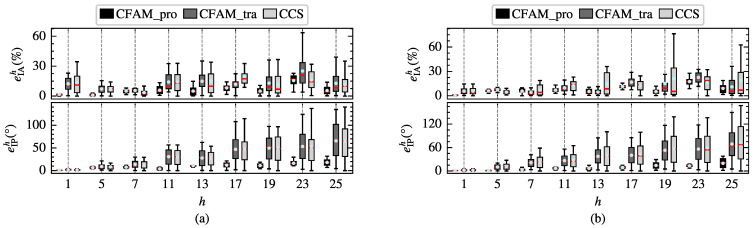
Distribution of the amplitude relative error $e_{\mathrm{IA}}^{h}$ and the phase absolute error $e_{\mathrm{IP}}^{h}$ of different order currents. (**a**) Intermittent load current condition. (**b**) Continuous load current condition.

To further compare the modeling performance of the harmonic models, the amplitude relative error $e_{\mathrm{IA}}^{h}$ and the phase absolute error $e_{\mathrm{IP}}^{h}$ of different order currents in the test samples are calculated separately. Figure 19 demonstrates the distribution of $e_{\mathrm{IA}}^{h}$ and $e_{\mathrm{IP}}^{h}$ for the three harmonic models under intermittent and continuous load current conditions. It can be observed that the mean errors and the width of the box plots for the analytical CFAM model calculated using the proposed method are generally smaller than those for the CCS model and the traditional CFAM model, indicating that the proposed method achieves higher modeling accuracy and narrower range of error fluctuations.

**Figure 20:**
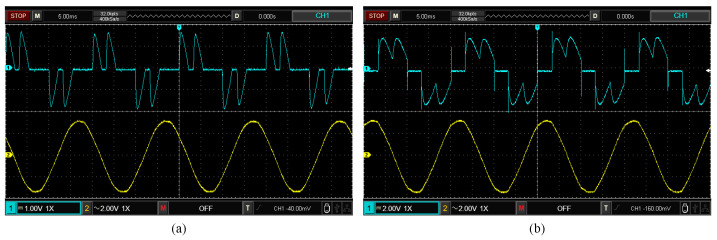
The measured waveforms of the AC−side resistor voltage (blue line) and the secondary winding line voltage (yellow line) under the two operating states of $S_{10}$ (**a**) and $S_{40}$ (**b**).

**Figure 21:**
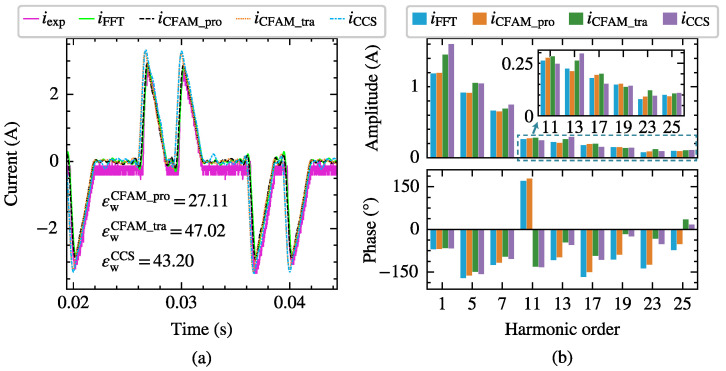
The current measurements and model estimates for the three-phase bridge rectifier under $S_{10}$ operating state. (**a**) Comparison of current waveforms. (**b**) Comparison of current amplitude spectrums and phase spectrums.

**Figure 22:**
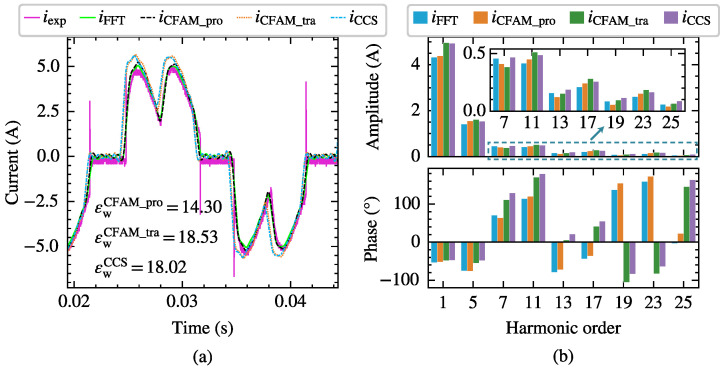
The current measurements and model estimates for the three-phase bridge rectifier under $S_{40}$ operating state. (**a**) Comparison of current waveforms. (**b**) Comparison of current amplitude spectrums and phase spectrums.

Figure 20a,b present the waveforms of the AC−side resistor voltage and the secondary winding line voltage measured by the oscilloscope under the two operating states of $S_{10}$ and $S_{40}$, respectively. Figure 21 and Figure 22 illustrate the waveforms and spectrum comparisons of the measured AC−side currents and model estimates for the rectifier under both operating states. Here, $i_{\exp}$ represents the experimentally measured current waveform, and $i_{\mathrm{FFT}}$ denotes the current waveform reconstructed from the fundamental and the first 25 characteristic harmonics of the measured current. The current waveform deviation coefficient $\varepsilon_{w}$ annotated in the figures indicates the waveform discrepancies between the estimated current and measured current. Comparison of the waveforms and spectrograms reveals that the currents calculated by the CFAM analytical model $i_{\mathrm{CFAM}\_\mathrm{pro}}$ are closer to the FFT analysis result $i_{\mathrm{FFT}}$, while the current $i_{\mathrm{CCS}}$ calculated by the CCS model and the current $i_{\mathrm{CFAM}\_\mathrm{tra}}$ calculated by the traditional CFAM model exhibit larger errors.

## 5. Conclusions

This paper proposes a calculation method for the CFAM analytical model of phase-controlled power electronic harmonic sources based on piecewise linearization. Phase-controlled power electronic equipment can be treated as a linear circuit in each individual operating stage, both before and after the conduction of the switching devices. Firstly, the time-domain current for each operating stage within one fundamental period is derived for any harmonic voltage at the PCC of the harmonic source. Subsequently, Fourier series are utilized to extract the harmonic current components, thereby constructing the CFAM analytical model of the harmonic source. Finally, taking single-phase and three-phase bridge rectifiers as examples, CFAM analytical models are established, respectively, under intermittent and continuous load current conditions, and the unified harmonic models for both conditions are further organized.

Based on MATLAB/Simulink software simulation and experimental tests, case studies on rectifier harmonic sources are conducted under different thyristor trigger angles and supply voltage distortions to verify the effectiveness of the proposed method. Extensive simulations with variable parameters are performed for both intermittent and continuous load current conditions of the single-phase and three-phase bridge rectifiers. Additionally, an experimental platform for the three-phase bridge rectifier is set up, and experimental tests are conducted under different thyristor trigger angles and load inductances. The proposed CFAM analytical model along with the traditional CFAM model and CCS model are used for harmonic source modeling, respectively, and the evaluation metrics are calculated based on the measured currents. The results from both simulations and experimental tests demonstrate that the CFAM analytical model provides higher accuracy and more stable performance for harmonic modeling.

The proposed calculation method of the CFAM analytical model is based on the piecewise linearization technique, which determines the operating stages within one fundamental period according to the conduction sequence of the switching devices. However, fully controlled equipment typically employs PWM technology to generate high-frequency pulses, causing the switching devices to rapidly turn on and off within one fundamental period, leading to numerous stage transitions. As a result, the proposed method struggles to calculate the CFAM analytical model for fully controlled equipment. In practical scenarios, accurately determining the equivalent resistance and inductance of circuits can be challenging. Future research will focus on the estimation of equivalent circuit parameters and further explore the application of the CFAM analytical model in harmonic power flow calculation and other related harmonic analyses.

## Figures and Tables

**Table 1:** The measured PCC voltages under six typical harmonic scenarios.

	Case 1		Case 2		Case 3		Case 4		Case 5		Case 6	
	Mag	Phs	Mag	Phs	Mag	Phs	Mag	Phs	Mag	Phs	Mag	Phs
	(V)	(°)	(V)	(°)	(V)	(°)	(V)	(°)	(V)	(°)	(V)	(°)
1st	230.6	0	231.1	0	225.7	0	226.1	0	222.8	0	208.5	0
3rd	0.6	−55	1.4	−60	3.8	−64	6.4	−58	7.8	−55	8.2	−70
5th	1.2	164	2.1	170	3.4	255	2.2	117	2.7	134	3.2	120
7th	1.1	−1	0.9	24	1.2	38	2.1	153	1.8	134	2.4	115
9th	0.7	−71	0.5	230	1.7	192	2.2	180	2.0	173	2.2	167
11th	0.3	−6	0.6	−19	2.5	−99	0.8	73	0.9	56	1.5	39
13th	0.1	125	0.8	128	0.8	−10	1.3	132	1.5	120	1.7	108
15th	0.3	114	0.4	−73	0.5	20	0.5	95	0.7	117	1.2	139
17th	0.2	252	0.4	60	1.2	−60	0.8	88	0.8	54	1.1	20
19th	0.2	−72	0.3	166	1.1	−55	0.6	108	0.5	73	0.9	38
$V_{\mathrm{THD}}\left(\%\right)$	0.84		1.29		2.81		3.39		4.01		4.72	

**Table 2:** Evaluation metrics of different harmonic source models under intermittent load current conditions for the single-phase bridge rectifier.

Model	$\mu_{{\bar{e}}_{\mathrm{IA}}^{h}}$ (%)	$\sigma_{{\bar{e}}_{\mathrm{IA}}^{h}}$ (%)	$\mu_{{\bar{e}}_{\mathrm{IP}}^{h}}$ (°)	$\sigma_{{\bar{e}}_{\mathrm{IP}}^{h}}$ (°)	$\mu_{\varepsilon_{w}}$ (%)	$\sigma_{\varepsilon_{w}}$ (%)
CFAM_pro	2.61	0.58	0.91	0.41	1.34	0.06
CFAM_tra	6.94	4.97	11.31	8.04	2.01	0.70
CCS	11.47	4.47	13.52	7.64	5.31	3.23

**Table 3:** Evaluation metrics of different harmonic source models under continuous load current conditions for the single-phase bridge rectifier.

Model	$\mu_{{\bar{e}}_{\mathrm{IA}}^{h}}$ (%)	$\sigma_{{\bar{e}}_{\mathrm{IA}}^{h}}$ (%)	$\mu_{{\bar{e}}_{\mathrm{IP}}^{h}}$ (°)	$\sigma_{{\bar{e}}_{\mathrm{IP}}^{h}}$ (°)	$\mu_{\varepsilon_{w}}$ (%)	$\sigma_{\varepsilon_{w}}$ (%)
CFAM_pro	0.68	0.34	3.67	2.25	8.70	0.32
CFAM_tra	4.89	3.61	11.32	9.77	9.53	1.18
CCS	5.86	4.13	11.39	9.71	10.81	2.11

**Table 4:** Voltage magnitudes and phases of six cases.

	Case 7		Case 8		Case 9		Case 10		Case 11		Case 12	
	Mag	Phs	Mag	Phs	Mag	Phs	Mag	Phs	Mag	Phs	Mag	Phs
	(V)	(°)	(V)	(°)	(V)	(°)	(V)	(°)	(V)	(°)	(V)	(°)
1st	218.7	0	217.6	0	216.3	0	214.7	0	213.2	0	196.0	0
5th	1.9	−40	3.4	−38	4.8	−37	6.6	−38	8.1	−36	9.7	−33
7th	0.4	152	0.6	153	0.8	154	1.1	150	1.4	151	2.0	158
11th	0.6	17	1.1	21	1.6	24	2.1	19	2.5	23	3.2	31
13th	0.3	214	0.5	219	0.6	−138	0.8	−144	1.1	−140	1.4	−130
17th	0.4	75	0.6	81	0.9	85	1.2	78	1.5	84	1.8	97
19th	0.2	−87	0.4	−80	0.5	−75	0.7	−83	0.8	−77	1.1	−63
23th	0.3	133	0.4	141	0.6	147	0.8	−223	1.0	−214	1.3	−197
25th	0.1	−28	0.3	−19	0.4	−13	0.6	−24	0.6	−15	0.8	4
$V_{\mathrm{THD}}\left(\%\right)$	0.97		1.72		2.46		3.37		4.17		5.51	

**Table 5:** Evaluation metrics results for the three-phase bridge rectifier in intermittent current conditions.

Model	$\mu_{{\bar{e}}_{\mathrm{IA}}^{h}}$ (%)	$\sigma_{{\bar{e}}_{\mathrm{IA}}^{h}}$ (%)	$\mu_{{\bar{e}}_{\mathrm{IP}}^{h}}$ (°)	$\sigma_{{\bar{e}}_{\mathrm{IP}}^{h}}$ (°)	$\mu_{\varepsilon_{w}}$ (%)	$\sigma_{\varepsilon_{w}}$ (%)
CFAM_pro	2.44	0.36	5.59	0.49	13.74	0.97
CFAM_tra	6.81	4.81	17.33	12.98	17.85	5.56
CCS	8.33	5.26	17.49	12.92	19.76	6.84

**Table 6:** Evaluation metrics results for the three-phase bridge rectifier in continuous current conditions.

Model	$\mu_{{\bar{e}}_{\mathrm{IA}}^{h}}$ (%)	$\sigma_{{\bar{e}}_{\mathrm{IA}}^{h}}$ (%)	$\mu_{{\bar{e}}_{\mathrm{IP}}^{h}}$ (°)	$\sigma_{{\bar{e}}_{\mathrm{IP}}^{h}}$ (°)	$\mu_{\varepsilon_{w}}$ (%)	$\sigma_{\varepsilon_{w}}$ (%)
CFAM_pro	5.20	1.46	3.19	2.28	14.63	0.20
CFAM_tra	13.80	14.04	10.83	9.58	17.89	5.09
CCS	15.60	15.98	10.89	9.61	19.28	6.52

**Table 7:** Circuit parameter settings and DC side voltage measurements under intermittent load current conditions for the three-phase bridge rectifier.

	$S_{1}$	$S_{2}$	$S_{3}$	$S_{4}$	$S_{5}$	$S_{6}$	$S_{7}$	$S_{8}$	$S_{9}$	$S_{10}$
$\alpha \left({}^{\circ}\right)$	75	76	77	78	79	80	81	82	83	84
$L\left(\mathrm{mH}\right)$	1	1	1	1	1	1	1	1	1	1
$V_{\mathrm{dc}}\left(V\right)$	11.22	10.20	9.82	9.37	8.94	8.55	8.12	7.76	7.40	6.98
	$S_{11}$	$S_{12}$	$S_{13}$	$S_{14}$	$S_{15}$	$S_{16}$	$S_{17}$	$S_{18}$	$S_{19}$	$S_{20}$
$\alpha \left({}^{\circ}\right)$	75	76	77	78	79	80	81	82	83	84
$L\left(\mathrm{mH}\right)$	3	3	3	3	3	3	3	3	3	3
$V_{\mathrm{dc}}\left(V\right)$	10.44	9.95	9.53	9.06	8.66	8.24	7.92	7.47	7.06	6.68

**Table 8:** Circuit parameter settings and DC side voltage measurements under continuous load current conditions for the three-phase bridge rectifier.

	$S_{21}$	$S_{22}$	$S_{23}$	$S_{24}$	$S_{25}$	$S_{26}$	$S_{27}$	$S_{28}$	$S_{29}$	$S_{30}$
$\alpha \left({}^{\circ}\right)$	46	47	48	49	50	51	52	53	54	55
$L\left(\mathrm{mH}\right)$	1	1	1	1	1	1	1	1	1	1
$V_{\mathrm{dc}}\left(V\right)$	28.14	27.41	26.88	26.36	25.87	25.40	24.85	24.27	23.85	23.13
	$S_{31}$	$S_{32}$	$S_{33}$	$S_{34}$	$S_{35}$	$S_{36}$	$S_{37}$	$S_{38}$	$S_{39}$	$S_{40}$
$\alpha \left({}^{\circ}\right)$	46	47	48	49	50	51	52	53	54	55
$L\left(\mathrm{mH}\right)$	3	3	3	3	3	3	3	3	3	3
$V_{\mathrm{dc}}\left(V\right)$	27.97	27.31	26.84	26.52	25.80	25.31	24.84	24.30	23.80	23.15

**Table 9:** Evaluation metrics of the harmonic models under intermittent load current conditions for the three-phase bridge rectifier.

Model	$\mu_{{\bar{e}}_{\mathrm{IA}}^{h}}$ (%)	$\sigma_{{\bar{e}}_{\mathrm{IA}}^{h}}$ (%)	$\mu_{{\bar{e}}_{\mathrm{IP}}^{h}}$ (°)	$\sigma_{{\bar{e}}_{\mathrm{IP}}^{h}}$ (°)	$\mu_{\varepsilon_{w}}$ (%)	$\sigma_{\varepsilon_{w}}$ (%)
CFAM_pro	5.88	1.14	9.75	3.26	21.31	6.11
CFAM_tra	12.97	5.47	33.20	18.97	28.69	9.22
CCS	12.44	6.10	32.99	19.86	27.00	9.33

**Table 10:** Evaluation metrics of the harmonic models under continuous load current conditions for the three-phase bridge rectifier.

Model	$\mu_{{\bar{e}}_{\mathrm{IA}}^{h}}$ (%)	$\sigma_{{\bar{e}}_{\mathrm{IA}}^{h}}$ (%)	$\mu_{{\bar{e}}_{\mathrm{IP}}^{h}}$ (°)	$\sigma_{{\bar{e}}_{\mathrm{IP}}^{h}}$ (°)	$\mu_{\varepsilon_{w}}$ (%)	$\sigma_{\varepsilon_{w}}$ (%)
CFAM_pro	7.54	1.08	8.41	3.05	18.64	5.58
CFAM_tra	10.81	5.06	35.17	20.29	19.55	5.06
CCS	12.60	7.33	37.32	25.36	19.34	5.26

## Data Availability

The data presented in this study are available on request from the corresponding author.
